# The Polygenic and Monogenic Basis of Blood Traits and Diseases

**DOI:** 10.1016/j.cell.2020.08.008

**Published:** 2020-09-03

**Authors:** Dragana Vuckovic, Erik L. Bao, Parsa Akbari, Caleb A. Lareau, Abdou Mousas, Tao Jiang, Ming-Huei Chen, Laura M. Raffield, Manuel Tardaguila, Jennifer E. Huffman, Scott C. Ritchie, Karyn Megy, Hannes Ponstingl, Christopher J. Penkett, Patrick K. Albers, Emilie M. Wigdor, Saori Sakaue, Arden Moscati, Regina Manansala, Ken Sin Lo, Huijun Qian, Masato Akiyama, Traci M. Bartz, Yoav Ben-Shlomo, Andrew Beswick, Jette Bork-Jensen, Erwin P. Bottinger, Jennifer A. Brody, Frank J.A. van Rooij, Kumaraswamy N. Chitrala, Peter W.F. Wilson, Hélène Choquet, John Danesh, Emanuele Di Angelantonio, Niki Dimou, Jingzhong Ding, Paul Elliott, Tõnu Esko, Michele K. Evans, Stephan B. Felix, James S. Floyd, Linda Broer, Niels Grarup, Michael H. Guo, Qi Guo, Andreas Greinacher, Jeff Haessler, Torben Hansen, Joanna M.M. Howson, Wei Huang, Eric Jorgenson, Tim Kacprowski, Mika Kähönen, Yoichiro Kamatani, Masahiro Kanai, Savita Karthikeyan, Fotios Koskeridis, Leslie A. Lange, Terho Lehtimäki, Allan Linneberg, Yongmei Liu, Leo-Pekka Lyytikäinen, Ani Manichaikul, Koichi Matsuda, Karen L. Mohlke, Nina Mononen, Yoshinori Murakami, Girish N. Nadkarni, Kjell Nikus, Nathan Pankratz, Oluf Pedersen, Michael Preuss, Bruce M. Psaty, Olli T. Raitakari, Stephen S. Rich, Benjamin A.T. Rodriguez, Jonathan D. Rosen, Jerome I. Rotter, Petra Schubert, Cassandra N. Spracklen, Praveen Surendran, Hua Tang, Jean-Claude Tardif, Mohsen Ghanbari, Uwe Völker, Henry Völzke, Nicholas A. Watkins, Stefan Weiss, VA Million Veteran Program, Na Cai, Kousik Kundu, Stephen B. Watt, Klaudia Walter, Alan B. Zonderman, Kelly Cho, Yun Li, Ruth J.F. Loos, Julian C. Knight, Michel Georges, Oliver Stegle, Evangelos Evangelou, Yukinori Okada, David J. Roberts, Michael Inouye, Andrew D. Johnson, Paul L. Auer, William J. Astle, Alexander P. Reiner, Adam S. Butterworth, Willem H. Ouwehand, Guillaume Lettre, Vijay G. Sankaran, Nicole Soranzo

**Affiliations:** 1 Human Genetics, Wellcome Sanger Institute, Hinxton, CB10 1SA, UK; 2 National Institute for Health Research Blood and Transplant Research Unit (NIHR BTRU) in Donor Health and Genomics, University of Cambridge, Cambridge, CB1 8RN, UK; 3 Department of Epidemiology, University of Washington, Seattle, WA, 98109, USA; 4 Division of Hematology/Oncology, Boston Children’s Hospital and Department of Pediatric Oncology, Dana-Farber Cancer Institute, Harvard Medical School, Boston, MA, 02115, USA; 5 Broad Institute of MIT and Harvard, Cambridge, MA, 02142, USA; 6 Harvard-MIT Health Sciences and Technology, Harvard Medical School, Boston, MA, 02142, USA; 7 Department of Public Health and Primary Care, British Heart Foundation Cardiovascular Epidemiology Unit, University of Cambridge, Cambridge, CB1 8RN, UK; 8 MRC Biostatistics Unit, University of Cambridge, Cambridge, CB2 0SR, UK; 9 Montreal Heart Institute, Montreal, Quebec, H1T 1C8, Canada; 10 National Institute for Health Research Cambridge Biomedical Research Centre, University of Cambridge and Cambridge University Hospitals, Cambridge, CB2 0QQ, UK; 11 The Framingham Heart Study, National Heart, Lung and Blood Institute, Framingham, MA, 01702, USA; 12 Population Sciences Branch, Division of Intramural Research, National Heart, Lung and Blood Institute, Framingham, MA, 01702, USA; 13 Department of Genetics, University of North Carolina, Chapel Hill, NC, 27599, USA; 14 Center for Population Genomics, Massachusetts Veterans Epidemiology Research and Information Center (MAVERIC), VA Boston Healthcare System, Boston, MA, 02130, USA; 15 Department of Public Health and Primary Care, Cambridge Baker Systems Genomics Initiative, University of Cambridge, Cambridge, CB1 8RN, UK; 16 Cambridge Baker Systems Genomics Initiative, Baker Heart and Diabetes Institute, Melbourne, Victoria, VIC 3004, Australia; 17 British Heart Foundation Centre of Excellence, Division of Cardiovascular Medicine, Addenbrooke’s Hospital, Cambridge, CB2 0QQ, UK; 18 Department of Haematology, University of Cambridge, Cambridge, CB2 0PT, UK; 19 National Institute for Health Research (NIHR) BioResource, Cambridge University Hospitals, Cambridge, CB2 0PT, UK; 20 National Health Service (NHS) Blood and Transplant, Cambridge Biomedical Campus, Cambridge, CB2 0PT, UK; 21 Department of Statistical Genetics, Osaka University Graduate School of Medicine, Suita, Osaka, 565-0871, Japan; 22 Laboratory for Statistical Analysis, RIKEN Center for Integrative Medical Sciences, Yokohama, Kanagawa, 230-0045, Japan; 23 Icahn School of Medicine at Mount Sinai, The Charles Bronfman Institute for Personalized Medicine, New York, NY, 10029, USA; 24 Zilber School of Public Health, University of Wisconsin-Milwaukee, Milwaukee, WI, 53201, USA; 25 Department of Statistics and Operation Research, University of North Carolina, Chapel Hill, NC, 27599, USA; 26 Department of Ocular Pathology and Imaging Science, Graduate School of Medical Sciences, Kyushu University, Fukuoka, 812-8581, Japan; 27 Department of Biostatistics, University of Washington, Seattle, WA, 98101, USA; 28 Population Health Sciences, Bristol Medical School, University of Bristol, Bristol, BS8 1QU, UK; 29 Translational Health Sciences, Musculoskeletal Research Unit, Bristol Medical School, University of Bristol, Bristol, BS10 5NB, UK; 30 Novo Nordisk Foundation Center for Basic Metabolic Research, Faculty of Health and Medical Sciences, University of Copenhagen, Copenhagen, 2200, Denmark; 31 Hasso-Plattner-Institut, Universität Potsdam, Potsdam, 14469, Germany; 32 Department of Medicine, University of Washington, Seattle, WA, 98101, USA; 33 Department of Epidemiology, Erasmus University Medical Center Rotterdam, Rotterdam, 3015 GE, the Netherlands; 34 Laboratory of Epidemiology and Population Science, National Institute on Aging/NIH, Baltimore, MD, 21224, USA; 35 Atlanta VA Medical Center, Decatur, GA, 30033, USA; 36 Division of Research, Kaiser Permanente Northern California, Oakland, CA, 94612, USA; 37 Health Data Research UK Cambridge, Wellcome Genome Campus and University of Cambridge, Cambridge, CB10 1SA, UK; 38 British Heart Foundation Centre of Research Excellence, University of Cambridge, Cambridge, CB1 8RN, UK; 39 Section of Nutrition and Metabolism, International Agency for Research on Cancer, Lyon, 69008, France; 40 Department of Hygiene and Epidemiology, University of Ioannina Medical School, Ioannina, 45110, Greece; 41 Department of Internal Medicine, Section of Gerontology and Geriatric Medicine, Wake Forest School of Medicine, Winston-Salem, NC, 27101, USA; 42 Department of Epidemiology and Biostatistics, Imperial College London, London, W2 1PG, UK; 43 Imperial Biomedical Research Centre, Imperial College London and Imperial College NHS Healthcare Trust, London, W2 1NY, UK; 44 Medical Research Council Centre for Environment and Health, Imperial College London, London, W2 1PG, UK; 45 UK Dementia Research Institute, Imperial College London, London, WC1E 6BT, UK; 46 Health Data Research UK London, London, W2 1PG, UK; 47 Department of Internal Medicine B, University Medicine Greifswald, Greifswald, 17475, Germany; 48 German Center for Cardiovascular Research (DZHK), Partner Site Greifswald, Greifswald, 17475, Germany; 49 Department of Epidemiology, University of Washington, Seattle, WA, 98101, USA; 50 Department of Internal Medicine, Erasmus University Medical Center Rotterdam, Rotterdam, 3015 GE, the Netherlands; 51 Department of Neurology, University of Pennsylvania, Philadelphia, PA, 19104, USA; 52 Institute for Immunology and Transfusion Medicine, University Medicine Greifswald, Greifswald, 17475, Germany; 53 Division of Public Health Sciences, Fred Hutchinson Cancer Research Center, Seattle, WA, 98101, USA; 54 Novo Nordisk Research Centre Oxford, Oxford, OX3 7FZ, UK; 55 Department of Genetics, Shanghai-MOST Key Laboratory of Health and Disease Genomics, Chinese National Human Genome Center and Shanghai Industrial Technology Institute (SITI), Shanghai, 201203, China; 56 Interfaculty Institute of Genetics and Functional Genomics, University Medicine Greifswald, Greifswald, 17475, Germany; 57 Chair of Experimental Bioinformatics, Research Group Computational Systems Medicine, Technical University of Munich, Freising-Weihenstephan, 85354, Germany; 58 Department of Clinical Physiology, Tampere University Hospital, Tampere, 33521, Finland; 59 Department of Clinical Physiology, Finnish Cardiovascular Research Center - Tampere, Faculty of Medicine and Health Technology, Tampere University, Tampere, 33014, Finland; 60 Laboratory of Complex Trait Genomics, Department of Computational Biology and Medical Sciences, Graduate School of Frontier Sciences, The University of Tokyo, Tokyo, 108-8639, Japan; 61 Analytic and Translational Genetics Unit, Massachusetts General Hospital, Boston, MA, 02114, USA; 62 Department of Medicine, University of Colorado Denver, Anschutz Medical Campus, Aurora, CO, 80045, USA; 63 Department of Clinical Chemistry, Fimlab Laboratories, Tampere, 33520, Finland; 64 Department of Clinical Chemistry, Finnish Cardiovascular Research Center - Tampere, Faculty of Medicine and Health Technology, Tampere University, Tampere, 33014, Finland; 65 Center for Clinical Research and Prevention, Bispebjerg and Frederiksberg Hospital, Frederiksberg, 2000, Denmark; 66 Department of Clinical Medicine, Faculty of Health and Medical Sciences, University of Copenhagen, Copenhagen, 2200, Denmark; 67 Department of Medicine, Division of Cardiology, Duke Molecular Physiology Institute, Duke University Medical Center, Durham, NC, 27701, USA; 68 Center for Public Health Genomics, University of Virginia, Charlottesville, VA, 22903, USA; 69 Department of Computational Biology and Medical Sciences, Graduate school of Frontier Sciences, The University of Tokyo, Tokyo, 108-8639, Japan; 70 Division of Molecular Pathology, The Institute of Medical Science, The University of Tokyo, Tokyo, 108-8639, Japan; 71 Department of Cardiology, Heart Center, Tampere University Hospital, Tampere, 33521, Finland; 72 Department of Cardiology, Finnish Cardiovascular Research Center - Tampere, Faculty of Medicine and Health Technology, Tampere University, Tampere, 33014, Finland; 73 Department of Laboratory Medicine and Pathology, University of Minnesota, Minneapolis, MN, 55455, USA; 74 Departments of Epidemiology, University of Washington, Seattle, WA, 98101, USA; 75 Department of Health Services, University of Washington, Seattle, WA, 98101, USA; 76 Kaiser Permanente Washington Health Research Institute, Seattle, WA, 98101, USA; 77 Centre for Population Health Research, University of Turku and Turku University Hospital, Turku, 20521, Finland; 78 Research Centre of Applied and Preventive Cardiovascular Medicine, University of Turku, Turku, 20521, Finland; 79 Department of Clinical Physiology and Nuclear Medicine, Turku University Hospital, Turku, 20521, Finland; 80 Department of Biostatistics, University of North Carolina, Chapel Hill, NC, 27599, USA; 81 Department of Pediatrics, The Institute for Translational Genomics and Population Sciences, The Lundquist Institute for Biomedical Innovation (formerly Los Angeles Biomedical Research Institute) at Harbor-UCLA Medical Center, Torrance, CA, 90502, USA; 82 Massachusetts Veterans Epidemiology Research and Information Center (MAVERIC), VA Boston Healthcare System, Boston, MA, 02130, USA; 83 Department of Biostatistics and Epidemiology, University of Massachusetts-Amherst, Amherst, MA, 01002, USA; 84 Health Data Research UK Cambridge, Wellcome Sanger Institute, Hinxton, CB10 1SA, UK; 85 Department of Public Health and Primary Care, Rutherford Fund Fellow, University of Cambridge, Cambridge, CB1 8RN, UK; 86 Department of Genetics, Stanford University School of Medicine, Stanford, CA, 94305, USA; 87 Department of Medicine, Faculty of Medicine, Université de Montréal, Montreal, Quebec, H3T 1J4, Canada; 88 Department of Genetics, School of Medicine, Mashhad University of Medical Sciences, Mashhad, 9177948564, Iran; 89 Institute for Community Medicine, University Medicine Greifswald, Greifswald, 17475, Germany; 90 The Alan Turing Institute, London, NW1 2DB, UK; 91 Department of Medicine, Division on Aging, Brigham and Women’s Hospital, Boston, MA, 02115, USA; 92 Department of Medicine, Harvard Medical School, Boston, MA, 02115, USA; 93 Department of Computer Science, University of North Carolina, Chapel Hill, NC, 27599, USA; 94 Wellcome Centre for Human Genetics, University of Oxford, Oxford, OX3 7BN, UK; 95 Unit of Animal Genomics, GIGA-R & Faculty of Veterinary Medicine, University of Liège, Liège, B-4000, Belgium; 96 European Bioinformatics Institute, European Molecular Biology Laboratory, Hinxton, CB10 1SA, UK; 97 Laboratory of Statistical Immunology, Osaka University Graduate School of Medicine, Suita, Osaka, 565-0871, Japan; 98 BRC Haematology Theme and Radcliffe Department of Medicine, University of Oxford, John Radcliffe Hospital, Oxford, OX3 9DU, UK; 99 NHSBT Blood and Transplant - Oxford Center, John Radcliffe Hospital, Oxford, OX3 9BQ, UK

**Keywords:** blood, genetics, hematopoiesis, rare disease, polygenic risk, fine-mapping, splicing, UK Biobank, omnigenic, chromatin

## Abstract

Blood cells play essential roles in human health, underpinning physiological processes such as immunity, oxygen transport, and clotting, which when perturbed cause a significant global health burden. Here we integrate data from UK Biobank and a large-scale international collaborative effort, including data for 563,085 European ancestry participants, and discover 5,106 new genetic variants independently associated with 29 blood cell phenotypes covering a range of variation impacting hematopoiesis. We holistically characterize the genetic architecture of hematopoiesis, assess the relevance of the omnigenic model to blood cell phenotypes, delineate relevant hematopoietic cell states influenced by regulatory genetic variants and gene networks, identify novel splice-altering variants mediating the associations, and assess the polygenic prediction potential for blood traits and clinical disorders at the interface of complex and Mendelian genetics. These results show the power of large-scale blood cell trait GWAS to interrogate clinically meaningful variants across a wide allelic spectrum of human variation.

## Highlights

• Largest genome-wide association study of blood cell traits to date
• Empiric assessments of omnigenic and infinitesimal models of polygenic variation
• Functional insights into how genetic variants impact human hematopoiesis
• Assessment of the effect of polygenic trait scores upon blood diseases

## Graphical Abstract

**Figure:**
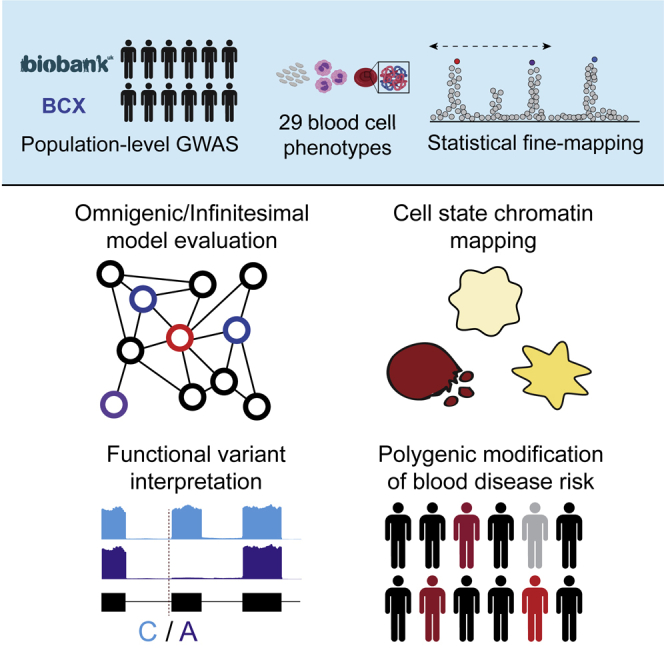

## Teaser

Analysis of blood cell traits in the UK Biobank and other cohorts illuminates the full genetic architecture of hematopoietic phenotypes, with evidence supporting the omnigenic model for complex traits and linking polygenic burden with monogenic blood diseases.

## Introduction

A major aspiration in human genetics is to understand how genetic variation impacts complex traits and diseases. Recent genome-wide association studies (GWAS) have identified thousands of genetic variants associated with complex phenotypes and provided insights into their genetic architecture. This has led to the recognition that complex trait heritability is polygenic, resulting from the cumulative effects of many genetic loci throughout the genome, each of modest effect size (Visscher et al., 2017; Timpson et al., 2018).

Hematopoiesis is a valuable paradigm for studying complex trait genetic architecture, since blood cell phenotypes are commonly measured in large population-based studies and the production of blood cells is a highly regulated, hierarchical, and intrinsic process that can be readily studied (Bao et al., 2019; Tardaguila and Soranzo, 2019). While there have been advances in understanding genetic loci associated with blood cell production, the spectrum of human genetic variation impacting hematopoiesis remains incompletely defined.

Most variants contributing to complex trait heritability are noncoding and located in genomic regulatory regions within relevant cell types. The availability of epigenomic and transcriptomic profiles for hematopoietic stem and progenitor and lineage-committed cells enable mechanistic dissection of the roles that different classes of genes have in hematopoiesis. Prior studies of blood cell traits have suggested that master transcription factors (TFs) may be impacted by genetic variation (Ulirsch et al., 2019), and it is likely that further studies may uncover additional roles for, and variation of, key hematopoietic regulators. Another priority is to advance understanding of network connectivity between trait-associated genes and variants, and this understanding can be informed by theoretical models. Recently, an “omnigenic” model has been proposed in which two types of genes (“core” versus “peripheral”) differentially contribute to complex trait heritability (Boyle et al., 2017; Liu et al., 2019). However, the extent to which the omnigenic model applies to various complex traits and diseases remains unclear and controversial (Wray et al., 2018).

Finally, although rare variants with large effects generally do not individually contribute substantially to overall complex trait variance, they can often highlight important biologic mechanisms and contribute to rare hematologic disorders, many of which are characterized by variable penetrance or expressivity. In addition, polygenic contributions of many variants with small effects can yield disease risk odds ratios comparable to or larger than that of known monogenic variants (Oetjens et al., 2019). Therefore, large population-based datasets can help to both reclassify the pathogenicity and penetrance of disease-associated variants, as well as understand the contribution of polygenic variation to the risk of blood diseases or as modifiers of rare variants that contribute to presumed monogenic blood disorders.

## Results

### Genetic Variants Associated with Blood Count Phenotypes

**Figure 1:**
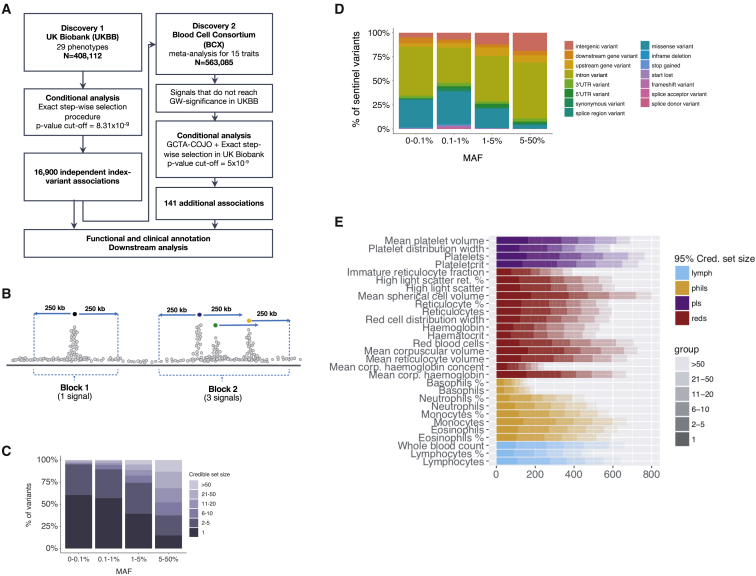
GWAS Study Design and Results (A–E) (A) Study design, (B) illustration for fine-mapping (FM) strategy showing how the FM blocks and the relevant number of causative signals were defined, (C) distribution of FM results by MAF, (D) distribution of FM results by sentinel annotation and MAF, and (E) FM 95% credible set size distribution for each sentinel, across all traits: different colors indicate different cell type groups.

**Figure S1:**
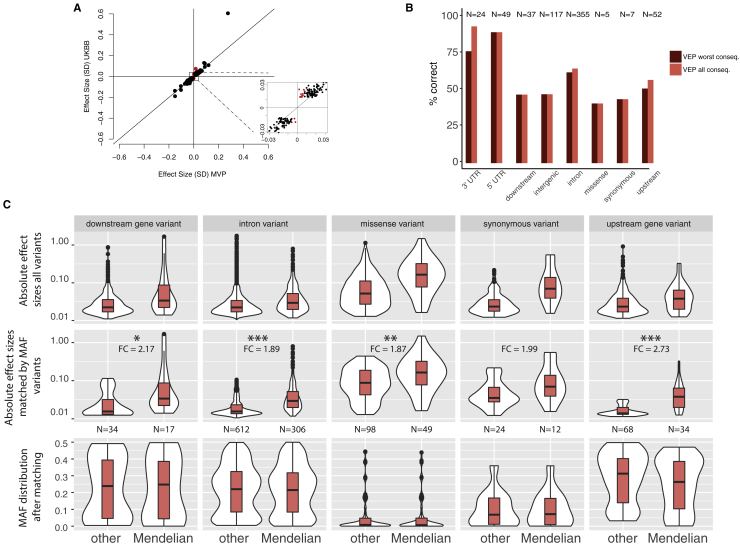
Replication and Mendelian Genes, Related to Figures 1 and 2 **A,** Comparison of replication effect size estimates, the x-axes shows effect sizes in MVP, the y-axes shows effect sizes in UK Biobank. The zoom-in panel highlights non-replicating variants in red. **B,** Proportions of correct gene to variant assignments for VEP worst consequence and VEP all consequences divided by functional annotation. Only known eQTLs in matched cell-types are shown and the correct gene is assumed to be the one identified by the eQTL experiment (eGene). **C,** Variants assigned by VEP to Mendelian genes across different functional annotations have higher effect sizes compared to other variants, after matching for MAF. The top 5 panels show absolute effect size distributions across all sentinel variants, where sentinels associated with multiple traits were included only once with the highest effect size. The middle 5 panels show the same distributions but after matching the non-Mendelian variants to the Mendelian ones by MAF. Stars denote significance: ^∗^ 0.005 < p value < 0.05; ^∗∗^ 0.0005 < p value < 0.005; ^∗∗∗^p value < 0.0005; FC = median fold change. The bottom 5 panels show the distributions of minor allele frequencies after matching.

We leveraged the power of the UK Biobank cohort to perform a genome-wide discovery analysis in N = 408,112 participants of European ancestry, investigating 29 blood cell phenotypes (Table S1). In parallel, we also performed tests for genetic associations with a subset of 15 phenotypes available in an additional 154,973 European ancestry participants from the Blood Cell Consortium (BCX) (Figure 1A, Table S2). A separate analysis of non-European participants is reported in a companion paper (Chen et al., 2020). Overall, this discovery effort identified 16,643 autosomal and 257 X-linked conditionally independent (Method Details) trait-variant associations from the first stage discovery and an additional 141 from the BCX meta-analysis (Tables S3 and S4). The 16,900 associations were assigned to 7,122 genomic loci (5,106 not described before) using a linkage disequilibrium (LD) clumping approach (Astle et al., 2016). Each locus was represented by a unique tag variant (between-tag pairwise LD r^2^ ≤ 0.8), and for simplicity, throughout the paper we use the term “sentinel variant” to refer to either a clump tag variant or a trait-specific conditionally independent signal. Overall, we nearly tripled the number of loci reported prior to this study (Astle et al., 2016). We assessed replication rates across three exemplar phenotypes (platelet count [PLT], lymphocyte count, and red blood cell count) for 210 variants on chromosome 1 in the Million Veteran Program (MVP, N = 271,280). We found that nearly all of them had directionally concordant effect size estimates (Pearson’s R^2^ = 0.94; Figure S1A), and 196 (93%) variants replicated at a nominal significance threshold (p < 0.05). The non-replicating ones exhibited similar effect sizes as in the discovery cohort but lacked power due to MVP having less than half the sample size of the discovery cohort (Figure S1A, zoom-in panel). Using a Bayesian method that accounts for multiple independent signals (Benner et al., 2016) (Method Details, Figure 1B), we fine-mapped 3,100 (19% of 16,643 autosomal) associations to a single putative causative variant (> 95% posterior probability [PP_FM_]) (Table S5), and more than half of the associated signals (n = 9,149, 55%) to fewer than 10 variants (Figure 1C). As expected, rare signals are more likely to be fine-mapped to smaller credible sets (Figure 1C). We assigned sentinels to genes using a stringent variant effect predictor (VEP) worst-consequence annotation (McLaren et al., 2016) to obtain a distribution of functional categories. Overall, 8,866 sentinels (83%) were annotated to a gene using this approach, of which 69% were intronic, 24% were in regulatory regions, and 7% were in protein-coding regions (5.5% non-synonymous and 1.5% synonymous; Figure 1D). The credible set size distribution (number of variants per credible set) was consistent across traits (Figure 1E).

### Genetic Architecture and Network Connectivity of Blood Cell Traits

**Figure 2:**
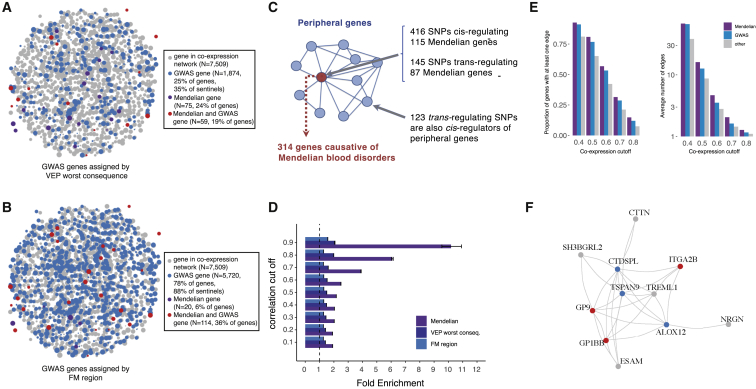
Network Connectivity (A–B) Coexpression network in whole blood. For illustrative purposes, a subset of highly coexpressed genes is shown (correlation > 0.7). Edges are omitted for clarity, and the node size summarizes the number and strength of coexpression links. Blue dots represent genes detected by GWAS, violet dots are Mendelian genes, and red dots show the intersection. Grey dots are genes in the coexpression network that do not belong to any of the previous categories. GWAS genes are defined by two different variant annotation approaches: VEP all consequences (A) and 500kb FM regions (B). (C) Diagram showing the hypothesized genetic architecture of healthy blood traits. At the core of the underlying molecular network is the set of Mendelian genes which cause blood disorders when mutated. Peripherally to the core lie regulatory genes which affect the phenotype through core genes. Cis and trans-eQTLs can give insights about cell-type specificity and can identify master regulators, i.e., genes that trans-regulate several core genes simultaneously. (D) Enrichment of sets of genes in the coexpression network at different correlation cut-offs. Whiskers indicate 95% CI for the fold enrichment estimate. (E) Proportion of network genes among Mendelian, GWAS, or other genes with > 1 edge, or average number of edges, at different correlation cut-offs. (F) Example of a sub-network containing 3 Mendelian genes involved in platelets (*GP9*, *ITGA2B*, *GP1BB*). As in (A), blue dots are GWAS genes, red dots are GWAS and previously known Mendelian genes, and gray dots are other coexpressed genes.

Hematopoiesis is a finely tuned process involving coordinated expression of hundreds of genes, and it is likely that a subset of the variants associated with peripheral blood cell counts and indices acts upon master regulators of this process. To identify whether genes discovered by GWAS identify networks of coregulated genes, we accessed a published coexpression network of 7,509 protein-coding genes expressed in whole blood (Nath et al., 2017) (Figures 2A and 2B; Method Details). Under the stringent VEP worst-consequence criteria used earlier, 25% of network genes (n = 1,874 genes) were annotated to a GWAS signal. A more permissive VEP any-consequence criteria annotated an additional 2.5% (27.5%, n = 2,070) genes. When all genes in the fine-mapping regions were considered (± 250-kb window), 78% of network genes could be linked to a GWAS locus, and 88% of sentinels were in proximity (< 250 kb) to a network gene, suggesting that genes linked to association signals are likely to be coregulated. Where possible, gene assignments were also validated using colocalization (Giambartolomei et al., 2018) with (expression quantitative trait loci) cis-eQTLs derived from six trait-matched blood cell types (platelets n = 424; CD19^+^ B cells, CD8^+^ T cells, CD4^+^ T cells and CD15^+^ neutrophils n = 300; CD14^+^ monocytes n = 1,490). Across 667 colocalizing cis-eQTLs, eGenes matched VEP worst-consequence genes in 65% of the cases (Figure S1B) and were contained in fine-mapping regions in 97% of the cases (Kreuzhuber, 2019).

Biological networks are organized hierarchically (Ravasz et al., 2002; Ravasz and Barabási, 2003; Carlson et al., 2006). The recently proposed “omnigenic” model (Boyle et al., 2017; Liu et al., 2019) postulates that a small number of genes at the center (or “core”) of the network are directly implicated in diseases or phenotypes of interest, but the variants in these genes contribute only a small proportion of the overall trait heritability. Most of the trait heritability is attributable to a much larger number of “peripheral” gene variants with small effect sizes that contribute to subtler physiological perturbations of phenotypes through trans-regulatory effects on core genes. We thus sought to empirically test the main assumptions of the omnigenic model, compared to a more continuous “infinitesimal” model of disease heritability (Wray et al., 2018) in order to inform its utility for disease gene discovery. We accessed a manually curated list of genes causative for stem cell and myeloid disorders (SMD, 206 genes); bleeding, thrombotic, and platelet disorders (BPD, 104 genes); and bone-marrow failure (BMF) syndromes (80 genes; Table S7) (Turro et al., 2020). GWAS loci for blood cell indices tended to be strongly enriched in and near Mendelian blood disorder genes (by 2.1-fold, p = 1.9×10^−22^), a phenomenon already described for many complex traits (Gieger et al., 2011; Durand and Rappold, 2013; Flannick et al., 2016). We then asked whether these Mendelian genes had properties expected of core genes.

**Figure S2:**
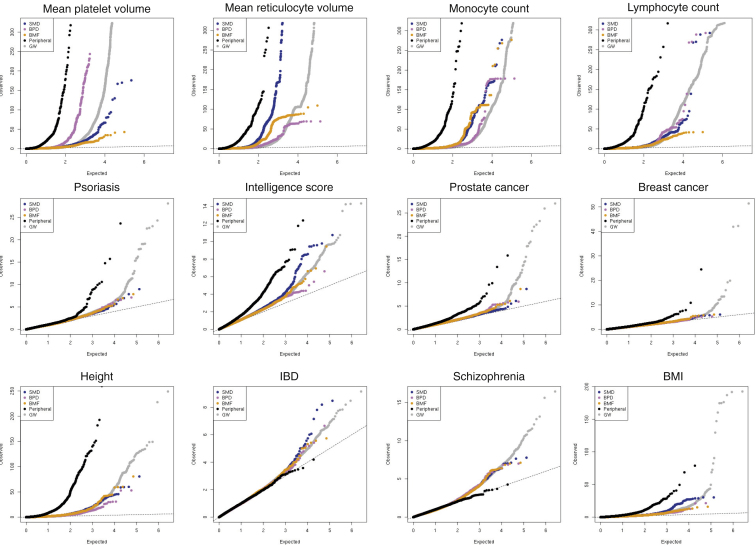
Mendelian and Peripheral Enrichment Q-Q Plots, Related to Figure 2 Each Q-Q plot shows the enrichment for variants assigned to a 100kb interval surrounding Mendelian genes. Different GWAS traits are included: 4 exemplar blood traits and 8 unrelated traits, selected to have at least 500 significant GWAS associations. Overall, with the exception of the “Intelligence” trait, most non-blood phenotypes do not show enrichment for variants mapped to Mendelian blood disorder genes. Conversely, peripheral associations were more likely to be enriched in non-blood traits, showing enrichment for six out of eight traits. SMD = stem cell and myeloid disorders, BPD = bleeding and thrombotic disorders, BMF = bone-marrow failure; GW = genome-wide.

A first assumption of the model is that core genes are strongly enriched at the center of biological networks (Figure 2C). Overall, we observed strong enrichments of both GWAS (fold enrichment [FE] = 1.86, permutation p < 10^−4^) and Mendelian (i.e., core, FE = 3.86, p < 10^−4^) genes in the full blood coexpression network (Nath et al., 2017) compared to permuted sets of protein-coding genes of similar size (Figure 2D; Table S6). Importantly, Mendelian genes had more connections in the coexpression network compared to other (non-Mendelian) genes, consistent with a centrality scenario (valid for coexpression cut-offs at 0.4–0.8, p ranging from 4×10^−4^ to 0.02, Wilcoxon test; Figure 2E). Finally, the expression of Mendelian genes was more correlated with other Mendelian genes (median coexpression coefficient = 0.11) than random sets of genes (median = 0.095, p = 0.007 permutation test). A second assumption is that variants assigned to core genes have larger effect sizes than peripheral genes. When compared to variants of comparable minor allele frequency (MAF) assigned to other genes, variants assigned to Mendelian genes (including previously unreported ones) showed significantly higher absolute effect sizes across all functional categories tested (fold change ranging from 1.87- to 2.73-fold increase; Figure S1C). Third, core genes should be more phenotype-specific as opposed to peripheral associations which act as regulators and could be shared across different phenotypes. We show by quartile-quartile (Q-Q) plot enrichments that this pattern holds true for Mendelian versus peripheral blood traits associations in a selection of eight non-blood related traits (Figure S2).

**Figure S3:**
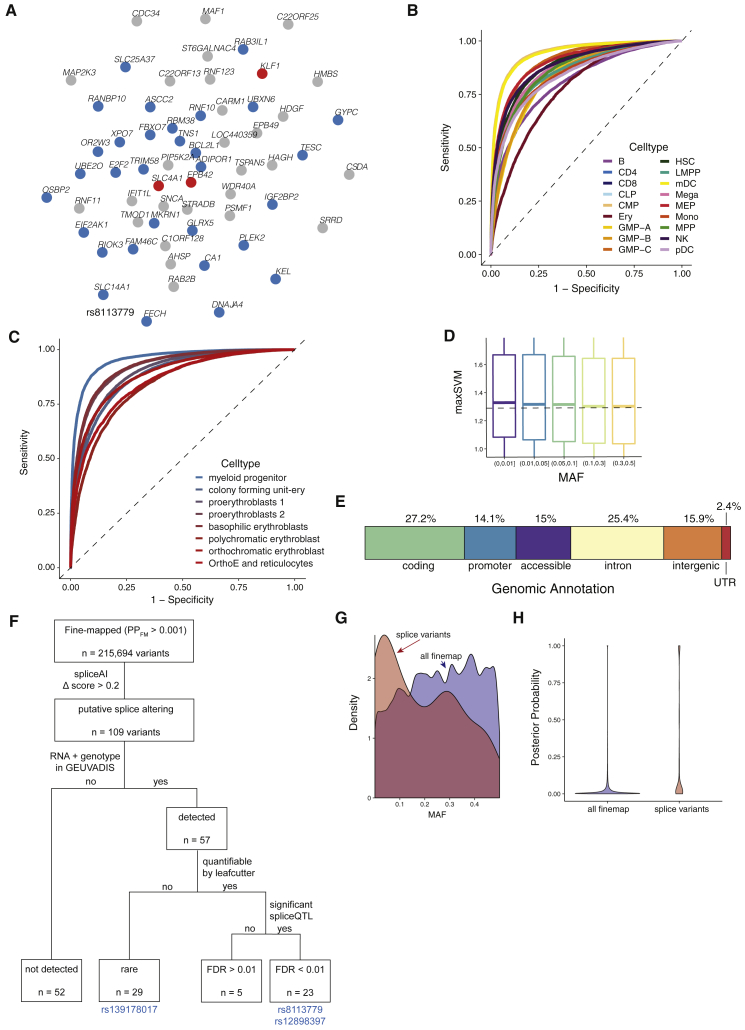
Network Examples and Functional Annotation, Related to Figure 2 **A,** A zoom-in example of the coexpression network, including connected genes with a very high correlation cut-off (0.8). Blue dots represent genes detected by GWAS, according to VEP worst-consequence annotation, red dots represent GWAS genes that are also Mendelian genes for blood disorders. Three Mendelian genes are identified, all of them involved in spherocytosis and other red-cell disorders. **B-C,** Receiver operating characteristic (ROC) curves for measuring classification performance of deltaSVM in two datasets: B) 18 hematopoietic populations sorted from bone marrow, and C) 8 stages of primary erythroid differentiation. **D**, Association between variant absolute deltaSVM score (maxSVM), reflecting a variant’s predicted disruption of chromatin accessibility, and bins of MAF. Dotted line indicates the median maxSVM score for the MAF 0.3-0.5 bin. **E,** Rare variants (MAC > 20, MAF < 1%, PP_FM_ > 0.50, conditionally independent) grouped by genomic annotation. **F,** Flow-chart depicting the steps involved in the identification and validation of blood trait-associated splice variants. **G,** Density distribution of variant MAF, comparing 109 putative splice variants to all fine-mapped blood trait variants. **H**, Violin plot of the fine-mapped posterior probability (PP_FM_) for putative splice variants versus all fine-mapped variants. For variants fine-mapped to multiple blood traits, we used the maximum PP_FM_.

The model also predicts that peripheral variants explain a large proportion of trait heritability through trans-regulation of core genes (Liu et al., 2019). To test this hypothesis, we accessed a large set of recently reported blood trans-eQTLs (Võsa et al., 2018). Mendelian genes were strongly enriched as targets of trans-eQTLs, compared to other GWAS genes (2.11-fold, Wilcoxon test, p = 4.7×10^−5^), after matching for expression levels and trans-eQTL Z-scores to account for differences in detection power, with the caveat that there may be other unaccounted factors involved. At a correlation cut-off of 0.8, a coexpression subnetwork of 26 GWAS-associated genes was centered on three known Mendelian genes causative for spherocytosis (*SLC4A1*, *EPB42*) and congenital anemia (*KLF1*; Figure S3A). Interestingly, these factors all play key roles in red blood cell cytoskeleton formation, a process regulated by *KLF1* (Ludwig et al., 2019). Another example is a subnetwork containing known platelet specific genes *GP9*, *GP1BB*, and *ITGA2B*, and eight other strongly coexpressed genes (Figure 2F). All of these genes are trans-regulated by the *ARHGEF3* gene, a known master regulator of megakaryopoiesis (Serbanovic-Canic et al., 2011). While these results are broadly compatible with expectations of the omnigenic model, first- and second-degree coexpression network neighbors of Mendelian genes were also enriched for GWAS associations (p < 1×10^−3^, permutation test) and thus had properties attributable to both core and peripheral genes. This indicates either that these loci may fit a more continuous infinitesimal model, or that our current proposed set of core genes is incomplete.

### Blood Cell Trait Variants Map to Lineage-Specific Hematopoietic Chromatin Landscapes

**Figure 3:**
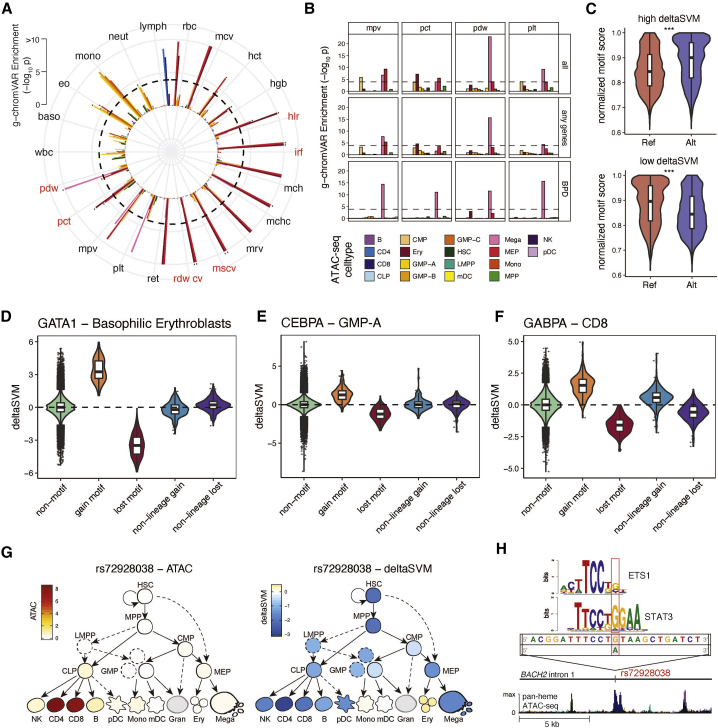
Functional Annotation of Blood Trait Variants (A) g-chromVAR results for FM variants (PP_FM_>0.1%) across 22 hematological traits. The Bonferroni-adjusted significance level (p = 0.05/22 traits ×18 cell types) is indicated by the dotted line. New traits are labeled in red. Novel enrichments are starred. The color legend for cell types is shared by panels (A), (B), and the trackplot in (H). mono = monocyte; gran = granulocyte; ery = erythroid; mega = megakaryocyte; CD4 = CD4+ T cell; CD8 = CD8+ T cell; B = B cell; NK = natural killer cell; mDC = myeloid dendritic cell; pDC, = plasmacytoid dendritic cell; MPP = multipotent progenitor; LMPP = lymphoid-primed multipotent progenitor; CMP = common myeloid progenitor; CLP = common lymphoid progenitor; GMP = granulocyte-macrophage progenitor; MEP = megakaryocyte–erythroid progenitor. (B) g-chromVAR enrichment results across 4 platelet traits (MPV, mean platelet volume; PCT, platelet crit; PDW, platelet distribution width; PLT, platelet count), using either all trait-associated variants (all), variants with any gene assignment (any gene), or only variants assigned to genes causative for BPD. The original Bonferroni-adjusted significance level is indicated by the dotted line. (C) The allelic effects of blood trait variants with (1) high (> 99th percentile) versus low (< 1st percentile) deltaSVM scores and (2) one or more predicted motif disruptions, on normalized motif scores. The normalized motif score represents the score for a variant-containing sequence as a percentage of the best score that motif could achieve on an ideal sequence. (D–F) Cell type-specific deltaSVM scores for variants disrupting the (D) GATA1, (E) CEBPA, or (F) GABPA motif compared to scores in non-motif-disrupting controls and non-lineage-specific cell types. Non-motif group indicates all other variants that do not disrupt the target TF. Gain or lost motif group contains variants predicted to create or disrupt the target TF motif, respectively, with the deltaSVM score for a lineage-specific cell type (erythroblast for GATA1, GMP for CEBPA, CD8 for GABPA). Non-lineage gain or lost indicates variants predicted to create or disrupt the target TF motif, but with the deltaSVM score for non-lineage-specific populations (CD8, CD4, and B cells for GATA1 and CEBPA; erythroblast and megakaryocytes for GABPA). (G) Lymphocyte count-associated variant rs72928038 has high chromatin accessibility (left) and deltaSVM score (right) in CD4 and CD8 populations. (H) rs72928038 is located within intron 1 of *BACH2*, and its minor allele A is predicted to break the motifs of TFs ETS1 and STAT3. In the bottom ATAC-seq plot, stacked colors represent accessibility for 18 hematopoietic cell types shown in (A).

We next sought to delineate relevant cell states impacted by core and peripheral gene networks. To this end, we integrated all fine-mapped (FM) variants (PP_FM_ > 0.1%) with chromatin accessibility profiles (ATAC-seq) of 18 human hematopoietic progenitor populations (Ulirsch et al., 2019). First, we noted that FM variants falling within hematopoietic open chromatin were strongly enriched in gene targets (assigned by VEP worst consequence) compared to non-accessible variants (OR = 1.4, Fisher’s p < 2.2×10^−16^), consistent with variants acting via trans-regulation of genes in hematopoietic cell states. Next, we used g-chromVAR, a high-resolution cell type enrichment method, to determine the hematopoietic populations most enriched for chromatin accessibility containing FM variants for 22 blood cell traits, including 6 new traits compared to a previous study in a smaller subset of the UK Biobank (Ulirsch et al., 2019). There were 43 lineage-specific enrichments surpassing experiment-wide significance (corrected for 18 cell types ×22 traits, p < 1.26×10^−4^) (Figure 3A), of which 20 were new, including novel enrichments in granulocyte-monocyte progenitor (GMP) cell subsets for variants regulating monocyte, eosinophil, and neutrophil counts.

We then wondered whether certain trait-cell type enrichments would strengthen when restricting to core genes for corresponding blood diseases. To this end, we calculated enrichments for four platelet traits, considering only the variants mapping to core genes for BPD. Whereas the gene-agnostic analysis produced significant enrichments in both megakaryocytes (n = 4) and its less differentiated myeloid precursors (n = 7), the core-gene restricted approach led to a strong signal for megakaryocytes (n = 4) but a lack of enrichment in any other population (Figure 3B). This suggests that in addition to their roles in Mendelian disease, core genes are also enriched for trans-regulatory variants acting specifically in their causal cell type.

Next, we sought to predict nucleotide-specific effects of variants on chromatin accessibility. We used deltaSVM, a support-vector machine classifier, to train genomic sequence features of the ATAC-seq from 18 hematopoietic cell populations (Figures S3B and S3C), and then applied the model to predict the allele-specific, cell type-specific impact of FM variants on chromatin accessibility (Lee et al., 2015). Out of 215,694 variants with PP_FM_ > 0.001 for one or more blood traits, we identified 22,152 variants with an absolute deltaSVM score above the 99th percentile for at least one hematopoietic cell type. Absolute deltaSVM score was negatively associated with MAF (linear regression p < 2.2×10^−16^) and positively associated with FM PP_FM_ (linear regression p = 1.0×10^−3^) (Figure S3D). Variants assigned to a gene by VEP worst consequence had stronger predicted effects on chromatin accessibility compared to intergenic variants (Student’s t test, p = 1.2×10^−3^); however, there was no significant difference in deltaSVM between variants assigned to “core” versus “peripheral” genes, suggesting that variant-mediated modulation of hematopoietic transcription occurs across the entire gene regulatory network rather than disproportionately impacting core genes.

To further characterize the regulatory effects of these variants, we predicted the potential for FM variants to disrupt 426 human TF motifs (Coetzee et al., 2015). Across motif-disrupting variants, alternative alleles predicted to increase chromatin accessibility (deltaSVM score > 99th percentile) had a significantly higher motif matching score compared to the reference allele (Method Details). The reverse was also true, indicating that deltaSVM scores track with the potential for variants to break or create TF motifs (Figure 3C). Moreover, this trend was cell type specific, as evidenced by the fact that variants affecting lineage-determining TFs had a higher deltaSVM score within lineage-specific cell types, such as GATA1-disrupting variants within erythroid progenitors (Wakabayashi et al., 2016), compared to other hematopoietic populations (Figures 3D–3F). We next sought to integrate these functional annotations in order to gain novel insights into biologically relevant variants. For example, variant rs72928038, previously identified in a locus associated with lymphocyte count (Astle et al., 2016), was fine-mapped here as the likely causal variant (PP_FM_ = 0.78), with the minor allele A (MAF = 18%) corresponding to decreased lymphocyte count. The variant maps to intron 1 of lymphoid TF *BACH2* (Richer et al., 2016) colocalizes with a H3K27ac histone QTL in CD4^+^ T cells (Kundu et al., 2020) and has high chromatin accessibility in the CD4^+^ and CD8^+^ T lymphoid populations. Interestingly, the lymphocyte count-decreasing minor allele has strongly negative deltaSVM scores (i.e., predicted to decrease chromatin accessibility) in the lymphoid lineage and is predicted to disrupt multiple TF motifs at the *BACH2* locus, including those with known roles in lymphocyte development, such as STAT3 and ETS1 (Figures 3G and 3H). This variant has been previously implicated in risk for several autoimmune conditions including rheumatoid arthritis (McAllister et al., 2013) and vitiligo (Jin et al., 2016). These lines of evidence suggest that rs72928038 may affect lymphocyte count by altering the binding of specific lymphoid TFs within T cell progenitors. Altogether, our functional characterization of non-coding blood trait variants highlights the value of incorporating lineage-specific chromatin accessibility profiles and motif disruption analyses to nominate high-confidence mechanisms.

### Clinical Impact of Rare Genetic Variants

**Figure 4:**
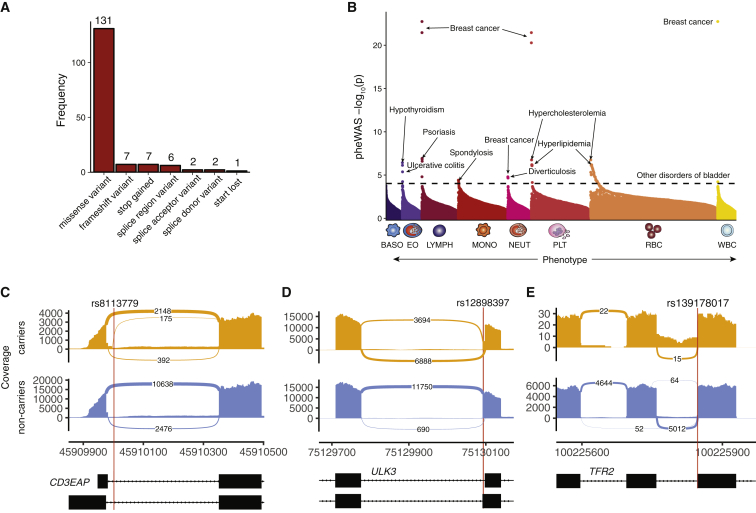
Characterization of Rare Blood Trait Variants (A) Distribution of coding consequences of 456 rare variants (MAC > 20, MAF < 1%), annotated using VEP. (B) Phenome-wide association study of these 456 rare variants across 529 well-represented clinical phenotypes in the UK Biobank (n up to 408,961). Variants are grouped by the hematopoietic lineage with which they are associated (BASO, basophil; EO, eosinophil; LYMPH, lymphocyte; MONO, monocyte; NEUT, neutrophil; PLT, platelet; RBC, red blood cell; WBC, white blood cell). Some variants appear in more than one category if they are associated with traits from distinct lineages. Text labels indicate the clinical outcomes with the strongest association per category. The dotted line denotes the Bonferroni-adjusted significance level (corrected for 529 phenotypes). (C–E) Sashimi plots depicting splice alterations at 3 loci as determined by RNA-sequencing analysis, comparing carriers of a specified blood trait variant (top track) versus non-carriers (bottom track). (C) Intronic donor gain splicing event in *CD3EAP* among carriers of rs8113779 (PP_FM_ = 0.23 for PLT, 2nd highest in credible set). Numbers within the splice junctions represent the number of reads supporting the junction. The x axis marks genomic coordinates. (D) Exonic donor gain splicing alteration in *ULK3* associated with rs12898397 (PP_FM_ = 0.071 for lymphocyte percent, 5th highest in credible set). (E) Donor loss splicing event in the *TFR2* locus, induced by variant rs139178017 (PP_FM_ = 0.73 for RDW, highest in credible set; PP_FM_ = 0.4 for MCV, 2nd highest in credible set).

The large sample size and dense imputation in this study gave us unprecedented statistical power to discover variants with low MAF and to assess their impact on human disease. First, we identified 574 rare (minor allele count [MAC] > 20, MAF < 1%) blood trait variants which were either conditionally independent lead variants and/or strongly fine-mapped (PP_FM_>0.5), of which 512 (89.2%) were previously unreported (Astle et al., 2016; Buniello et al., 2019). These variants had larger effect sizes (p < 2×10^−16^, t test) on blood traits as expected and were enriched for protein-coding consequences compared to other variants with similar PP_FM_ and/or lead conditional independence (27.2% versus 4.86%, χ^2^-test p < 2.2×10^−16^; Figure 4A, Figure S3E). Remarkably, these rare variants were strongly enriched for assignment to Mendelian blood genes (OR = 3.2, Fisher’s p = 4.22×10^−14^), even after excluding known pathogenic variants (Table 1; OR = 2.9, Fisher’s p = 4.46×10^−11^), but were not enriched for non-Mendelian genes (OR = 1.2, Fisher’s p = 0.18). These data support the hypothesis that a small group of high-effect rare variants disproportionately affect core genes for a complex trait.

**Table 1:** Annotation of Pathogenic Variants

Variant	Gene	AA change	Imputed/Genotyped	Disease (ICD10 code)	Incidence in UKBB (ICD10 codes/total N = 410,293)	Variant prevalence (carriers with disease/total) - matched to inheritance	Dominant/recessive pattern	GWAS blood phenotype	Pathogenic annotation db
rs113403872	*PKLR*	p.Arg110Gln	G	pyruvate kinase deficiency of red cells (D552)	7.3E-06	0/0	R	HCT, RBC, HGB	Clinvar, HGMD
rs116100695	*PKLR*	p.Arg486Trp	G	pyruvate kinase deficiency of red cells (D552)	7.3E-06	0/2	R	HGB, RET, RET%, IRF, HCT, RBC, HLR, HLR%	Clinvar, HGMD
rs61755431	*PKLR*	p.Arg569Gln	G	pyruvate kinase deficiency of red cells (D552)	7.3E-06	0/6	R	RET%	HGMD
rs35897051	*MPO*	c.2031-2A>C	I	myeloperoxidase deficiency (D7289)	0.00099441 (D728)	0/14	D	MONO, MONO%	Clinvar, HGMD
rs119468010	*MPO*	p.Arg569Trp	G	myeloperoxidase deficiency (D7289)	0.00099441 (D728)	0/4	D	MONO, MONO%	HGMD
rs1799945	*HFE*	p.His63Asp	G	haemochromatosis (E83119)	0.002371 (E831)	27/10,230	R	RET, RET%, MCHC, RDW	Clinvar, HGMD
rs1800730	*HFE*	p.Ser65Cys	I	haemochromatosis (E83119)	0.002371 (E831)	0/107	R	MCH, MCHC, MCV	Clinvar, HGMD
rs1800562	*HFE*	p.Cys282Tyr	I	haemochromatosis (E83119)	0.002371 (E831)	418/2,889	R	RET,RET%,MCV,RBC,MSCV,HGB,PLT,HCT,MCH,MCHC,RDW,PDW,MONO%,HLR,HLR%	HGMD
rs138156467	*CSF3R*	p.Trp547Ter	G	Neutropenia/philia (D709, D72828)	0.0119768 (D70), 0.0009944 (D728)	0/1	R	NEUT	HGMD
rs28928907	*MPL*	p.Arg102Pro	G	Congenital amegakaryocytic thrombocytopenia (D610)	5.1E-05	0/0	R	PCT	HGMD
rs33946267	*HBB*	p.Glu122Gln	I	Beta-thalassemia (D561)	1.7E-04	0/0	R	MCV	HGMD
rs61745086	*PIEZO1*	p.Pro2510Leu	I	Stomatocytosis dehydrated (D588)	4.9E-06	0/24	R	RET, RET%, HLR, HLR%, HCT, RBC, MCHC, HGB	HGMD
rs137853120	*TMPRSS6*	p.Asp521Asn	G	Iron-refractory iron deficiency anemia (IRIDA) (D508)	9.7E-03	0/0	R	MCV, MCH, RDW	HGMD
rs5030764	*GP9*	p.Asn61Ser	I	Bernard-Soulier Syndrome (D691)	4.1E-05	0/0	R	PDW, MPV, PLT, PCT	HGMD
rs41316003	*JAK2*	p.Arg1063Hist	G	Erythrocytosis (D750) with megakaryocytic atypia	9.0E-05	0/19	R	PCT, PLT	HGMD
rs146220228	*WAS*	p.Glu131Lys	G	X-linked thrombocytopenia, Wiskott-Aldrich syndrome (D820)	0	0/0	X	PDW	HGMD

The table shows sentinels that were annotated as pathogenic by either ClinVar or HGMD, using stringent criteria in each database. For each variant, we report the gene, the amino acid (AA) change caused, if the variant was genotyped or imputed, the associated disease, its incidence in UK Biobank, its prevalence among variant carriers (matched by disease inheritance, e.g., homozygous carriers are counted for recessive disorders), the phenotype associated by GWAS, and the database of origin (ClinVar or HGMD).

Given the large effects of these variants on blood traits, we next sought to test their pleiotropic associations with other clinical and disease traits. Thus, we performed a phenome-wide association study (PheWAS) for 456/574 rare variants using summary statistics for 529 well-represented clinical phenotypes from the UK Biobank cohort (https://www.leelabsg.org/resources) (Zhou et al., 2018). There were 112 significant associations involving 27 variants (Bonferroni-corrected p threshold of 9.45×10^−5^; Figure 4B, Table S8), of which 110 (98.2%) are currently unreported in the GWAS Catalog. Several biologically coherent associations stand out, including the missense variant rs78534766 in *ADCY7*, associated with autoimmune conditions (hypothyroidism, inflammatory bowel disease (Luo et al., 2017)) and eosinophil traits; several variants near *PIEZO1* associated with varicose veins (Fotiou et al., 2015; Van Hout et al., 2019) and erythroid traits; and a variant (rs45611741) in the 5′ UTR of *APOA5* associated with hypercholesterolemia (Nielsen et al., 2019) and Mean Corpuscular Volume (MCV), as well as Mean Corpuscular Hemoglobin Concentration (MCHC). Altogether, the PheWAS analysis revealed a variety of novel and relevant disease associations for rare blood trait variants and could point toward common mechanistic roles for these pleiotropic loci.

Splice-altering genetic variants are a prevalent and under-recognized class of variation underlying genetic disorders and complex trait regulation (Park et al., 2018). We hypothesized that a subset of blood trait variants, especially those that are rare with large effect sizes, may be mediated by splice alterations. We utilized a state-of-the-art neural net classifier, SpliceAI, to predict FM variants with splice-altering consequences (Jaganathan et al., 2019). The delta score has been shown to closely track with the validation rate of cryptic splice variants, thus approximating its splice-altering probability. Across 215,694 FM variants (PP_FM_>0.1%), we identified 109 variants with a putative splicing consequence in 106 unique genes (delta score > 0.2) (Figure S3F). Of these, 11 (10%) were rare (MAF < 1%) and confidently fine-mapped (PP_FM_>0.5; Table S9). Strikingly, 85% (93/109) of the variants, including 9/16 with delta score > 0.8, fell in non-canonical splice sites, meaning they lie outside the essential GT and AG splice junction dinucleotides. In addition, putative splice variants had lower MAF (Mann-Whitney U p = 5.08×10^−8^) and higher PP_FM_ (Mann-Whitney U p = 9.89×10^−6^) compared to other FM variants (Figures S3G and S3H). Even when matched by MAF and PP_FM,_ splice variants also had a 1.5-fold higher GWAS effect size (Mann-Whitney U p = 0.007). To validate these *in silico* predictions, we examined isoform variation in RNA-sequencing data of 465 participants from the Geuvadis project (Lappalainen et al., 2013) and used the LeafCutter tool to identify splicing quantitative trait loci (sQTLs) (Li et al., 2018). After excluding variants with insufficient statistical power in GEUVADIS, LeafCutter quantified differential splicing effects for 28/109 (26%) putative splice variants. Of these, 23/28 (82%) were identified as sQTLs at a 5% false discovery rate. For example, two common variants falling within 95% credible sets for PLT and lymphocyte count (rs8113779, MAF = 16% and rs12898397, MAF = 37%) were predicted to produce donor gain splice alterations in *CD3EAP* and *ULK3* respectively. These effects were validated by LeafCutter (rs8113779, adjusted p = 3.54×10^−47^; rs12898397, adjusted p = 3.39×10^−104^), with alternative splice sites produced by these variants in Geuvadis (Figures 4C and 4D). Finally, we highlight a previously unreported splice variant which was too rare to be quantified by LeafCutter but has interesting biological connections. rs139178017 (MAF = 0.53%) is a strongly FM variant in a novel association locus for red cell distribution width (RDW) (PP_FM_ = 0.73) and MCV (PP_FM_ = 0.4). It is predicted to induce a donor loss splice alteration for transferrin receptor 2 (*TFR2*), a partner of the erythropoietin receptor and a known regulator of erythropoiesis (Nai et al., 2015; Nandakumar et al., 2019). Compared to non-carriers, the 4 carriers of rs139178017 harbored substantially increased transcripts with intron retention adjacent to this variant (Figure 4E). These findings support the idea that large GWAS are well powered to identify splice variants with large phenotypic effects (Li et al., 2016), and these splice variants represent a currently under-appreciated mechanism of trait regulation in GWAS loci.

### Contribution of Polygenic Variation to Blood Cell Traits and Complex Human Diseases

**Figure 5:**
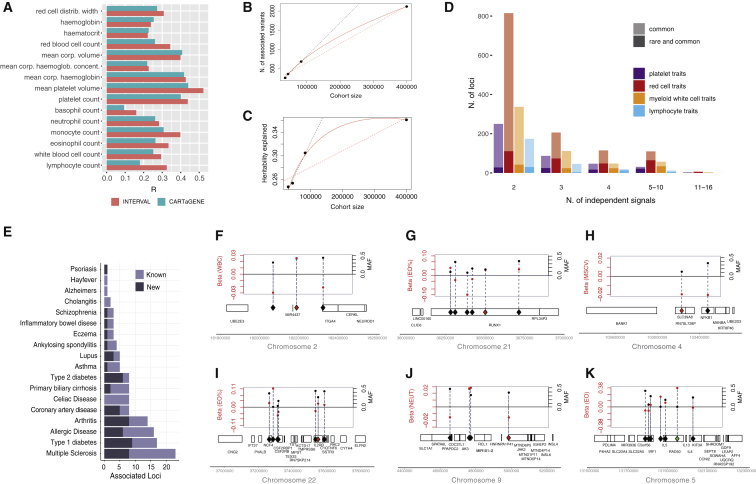
Polygenic Prediction of Blood Traits and Contribution to Common Diseases (A) Portability of the PGS across populations with European ancestry for 15 available traits. The red bar represents the Pearson’s correlation (R) between the score and the trait in the validation cohort (INTERVAL). Blue bars show the same in a French Canadian cohort called CARTAgENE. (B and C) Saturation analysis showing the number of discovered variants (B) and the proportion of heritability explained (C) as a function of GWAS sample size for mean platelet volume. The black dotted line is a linear projection of the first 3 points, the red dotted line is a linear interpolation of all points, and the red solid curve is the best model fitting the 4 points. (D) Number of loci with multiple sentinel variants, stratified by trait group. (E) Number of disease loci colocalizing (posterior probability > 99%) with at least one blood count locus, colored by known vs. new loci. (F–K) Examples of loci with multiple sentinels associated with blood cell counts, and with at least one disease-colocalization (red diamond) or PheWAS association (green diamond) for the following genes and diseases: *ITGA4* and Inflammatory Bowel Disease (IBD) (F), *RUNX1* and Rheumatoid Arthritis (G), *NFKB1* and IBD (H), *C1QTNF6* and Type-1 Diabetes (I), *JAK2* and IBD (J), *IL4* and asthma (K). In each panel, black dots show MAF (right y axis) and red dots show the effect size (in SD for the phenotype between brackets, left y axis) of each variant as a function of the variant’s position in the genomic interval.

Our study has identified the largest number of variants ever associated with a single group of correlated phenotypes. While each common (MAF ≥1%) variant accounts for a small effect, their joint effect may be substantial. We used different variant selection criteria to build weighted polygenic scores (PGSs) based on the UK Biobank study and selected the one yielding most predictive power for the 29 blood measurements in an independent cohort (INTERVAL study; Method Details; Table S10). Remarkably, PGS based on hundreds of common sentinel variants (135–689 depending on trait) were shown to be more predictive than larger SNP sets employing more liberal significance thresholds, in line with findings for autoimmune diseases (Abraham et al., 2014) but in contrast to other common human diseases (Khera et al., 2019). The proportion of phenotypic variance explained (R^2^) by the PGS ranged between 2.5% for basophil count to 27.3% for mean platelet volume. Estimates obtained for the same score in an independent cohort of 2,314 French Canadians (CARTaGENE) for 15 available traits were broadly comparable, confirming portability of the PGS between European-ancestry groups (Figure 5A). The causal relationship between genetic variants determining eosinophil count and asthma risk has been previously demonstrated (Astle et al., 2016). Focusing on this exemplar disease, we can show that the eosinophil count PGS was also significantly associated with asthma incidence in UK Biobank (odds ratio [OR] = 1.17, 95% confidence interval [CI] = 1.13–1.21, p = 1.02×10^−19^), suggesting the potential utility of PGSs for blood biomarkers in the clinic.

**Figure S4:**
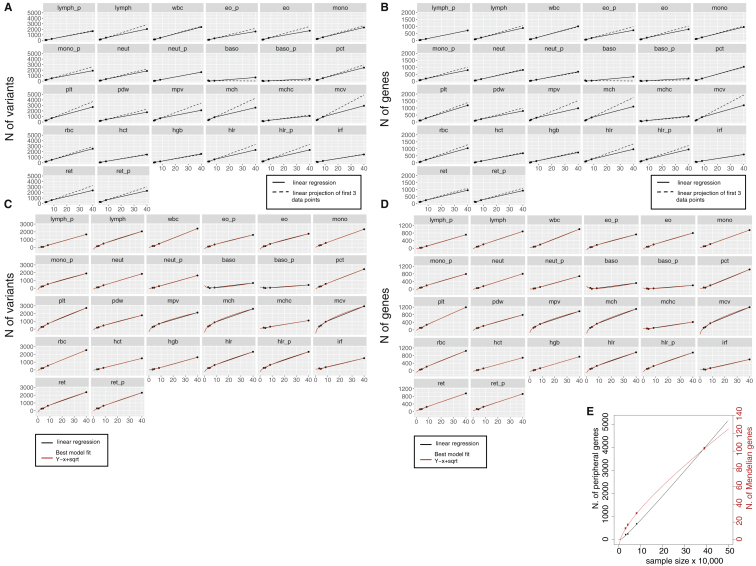
Saturation Models, Related to Figure 5 **A,** For each trait, we show the number of conditionally independent variants (y-axes) discovered by GWAS in four cohorts of increasing sample size. The sample size is shown on x-axes in 10,000 s. Two linear regression lines are shown: the full black line represents a regression including all 4 data points, the dotted black line represents a linear projection of the first three data points for comparison. A decreasing trend can be observed for almost all traits. **B,** Similarly to panel a, the number of GWAS-identified genes is shown on the y-axes. Genes were identified by VEP worst-consequence annotations. **C,** The same data points as in panel a are now shown with the best fitting model line in red, which correspond to a square-root growth model. **D,** The same data points as in panel b are now shown with the best fitting model line in red, which corresponds to a square-root growth model. **E,** The plot shows the saturation analysis of the number of discovered Mendelian genes (red color) and peripheral genes (black color) as a function of the discovery sample size. Both lines represent the best fitting model interpolating the dots and are defined as a function of the square-root of the sample size.

Intriguingly, the behavior of the PGSs suggests that the current discovery sample sizes may have achieved saturation of biological signals for blood cell traits. To begin to test this hypothesis, we modeled different discovery measures (total number of variants, loci, genes, and heritability explained) as a function of increasing discovery sample sizes. The best-fitting model shows a quadratic rate of discovery decrease across all tested measures and traits (Figures S4A–S4D). However, while the total numbers of associations detected does not seem to reach a plateau, the heritability explained does (Figures 5B and 5C), suggesting that GWAS with larger sample sizes will provide new discoveries, but of smaller and smaller effects, with the exception of unobserved rare variants. In line with the fact that variants assigned to Mendelian genes have higher effect sizes, these showed a faster saturation curve compared to other genes (Figure S4D). Larger independent discovery datasets will be required to conclusively validate this observation.

Finally, we wondered if multiple sentinels at a single locus could underlie associations with complex diseases and help define an allelic series at pharmacologically relevant genes. 20% of blood trait loci had ≥2 sentinels, including some unusually large sets (Figure 5D). We overlapped these regions with colocalization results for 18 common human diseases (Figure 5E). Figures 5F–5J show 6 instances of such conditionally independent variant sets, of which 3 involve a known drug target. For example, the type I diabetes (T1D) locus tagged by rs5845323 on chromosome 9 contains one rare and six common variants, all associated with eosinophil percentage (Figure 5I). While the colocalizing T1D variant is intronic in *C1QTNF6* gene, the coding-synonymous one from the series is in *IL2RB* (interleukin 2 receptor subunit beta). It has recently been proposed that the cancer drug Aldesleukin (recombinant IL-2, which binds IL2RB) may be repurposed to treat T1D at low doses, and the drug is currently in phase II clinical trial for this therapeutic application (Todd et al., 2016) (ClinicalTrials.gov ID: NCT01862120). There were 3 colocalizing loci between asthma and eosinophil count and/or percentage and a further three novel PheWAS associations of rare non-coding variants near known asthma genes (*GATA3*, *RAD50*, and *IL33).* One of the rare variants is part of a 270-kb set of sentinels on chromosome 5 associated with eosinophil count, including another rare variant and 5 common signals (Figure 5K). The genes implicated are *C5orf56* (*IRF1-AS1* or *IRF1* antisense RNA 1), *IRF1*, *IL5*, *RAD50*, *IL13*, *KIF3A*, and *IL4.* Interestingly, both IL5 and IL4 are current therapeutic targets for treating a number of allergic diseases (Ortega et al., 2014; Chang and Nadeau, 2017). Overall, this large set of conditionally independent variants informs future efforts to define allelic series to study genes of pharmacological importance (Claussnitzer et al., 2020).

### The Influence of Polygenic Variation on Blood Disorders

**Figure 6:**
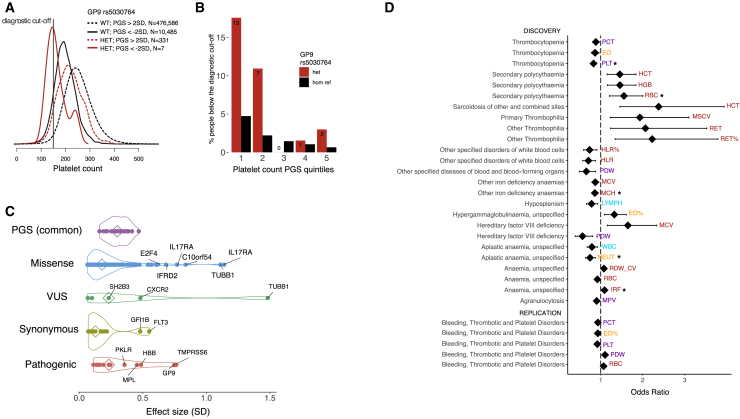
Contribution of Polygenic and Rare Variation to Blood Diseases (A) Density distribution of PLT (10^9^/liter) for UK Biobank participants who are heterozygous carriers (HET, red line) or wild-type (WT, black line) of the *GP9* rs5030764 c.182A>G (p.Asn61Ser) variant pathogenic for Bernard-Soulier syndrome, plotted for participants whose PGS is above or below 2 SDs of the population platelet PGS. (B) Proportion of participants below the normal range for PLT (150×10^9^/l) depending on PGS quintiles and *GP9* rs5030764 carriage status. (C) Absolute effect sizes comparison between different rare variant annotations and the common polygenic score. A subset of previously unreported missense variants shows high effect sizes comparable to known pathogenic ones, nominating them as putative new pathogenic candidates. The contribution of the polygenic score is comparable to that of a pathogenic variant in heterozygosity. Diamond shapes represent median values. (D) Forest plot showing the association of PGS with rare blood disorders, top 30 results (by p-value) are shown. Significant associations, after Bonferroni correction, are indicated by the ^∗^ symbol for the discovery stage, while replication effects shown are all nominally significant. Diamonds represent odds ratios and whiskers show the 95% confidence interval.

Mendelian blood disorders display considerable heterogeneity in penetrance and expressivity. Furthermore, estimates of effect size and penetrance of pathogenic variants tend to be inflated when ascertained from patient populations (Wright et al., 2019). While the PGSs defined by the common variants discovered in this study explain a substantial proportion of variance of respective phenotypes, the extent to which polygenic variation contributes to the manifestation of rare diseases remains to be determined. To address this question, we first explored the genetic landscape of classical blood disorders in UK Biobank. We annotated each protein-coding sentinel variant using (1) ClinVar (Landrum et al., 2014), (2) Human Gene Mutation Database (HGMD) (Stenson et al., 2017), and (3) a recently curated list of variants for rare blood disorders from the Rare Disease Pilot for the 100,000 Genomes Project (NIHR-RD) (Turro et al., 2020). Overall, 101 sentinels were included in one or more databases above, of which 80% were coding, 10% were annotated to 3′ or 5′ UTRs, and the remaining were splice or intronic variants. 16/101 (16%) were annotated to be pathogenic in either ClinVar or HGMD using strict criteria (Method Details), involving 11 genes (*PKLR*, *HFE*, *HBB*, *PIEZO1*, *TMPRSS6*, *JAK2*, *MPO*, *CSF3R*, *MPL*, *GP9*, and *WAS*; Table 1). Only 5/16 variants satisfied the pathogenicity criteria in both ClinVar and HGMD. Of these five, two variants previously reported as pathogenic for autosomal recessive diseases (rs116100695 in *PKLR* for pyruvate kinase deficiency of red cells and rs1800730 in *HFE* for hemochromatosis) were found in apparently healthy homozygous UK Biobank participants. Similarly, we found apparently healthy homozygous carriers for other four recessive variants, reported as pathogenic in HGMD, but not in ClinVar (rs61755431 in *PKLR* for pyruvate kinase deficiency or red cells, rs138156467 in *CSF3R* for neutropenia, rs61745086 in *PIEZO1* for dehydrated stomatocytosis and rs41316003 in *JAK2* for erythrocytosis and thrombocytosis). This lack of disease phenotype may be indicative of low penetrance, missing health record data, misannotation of the pathogenicity, or undiscovered compensatory effects either by rare variants or polygenic variation. For two additional recessive variants (rs137853120 in *TMPRSS6* for iron-refractory iron deficiency anemia and rs5030764 in *GP9* for Bernard-Soulier Syndrome) we observed no homozygous carriers, but heterozygous carriers were around 3 times more likely to have blood indices outside the normal range (hemoglobin < 12 g/dl, PLT < 150×10^9^/l), demonstrating previously unreported dosage-dependent effects (OR = 3.25, 95% CI = 1.85, 5.37, p = 5×10^−5^ [Figures 6A and 6B] and OR = 3.79, 95% CI = 2.40–5.68, p = 1.1×10^−9^, respectively). Data were inconclusive for the remaining 8 variants, either because there were no homozygous carriers in UK Biobank (rs113403872 in *PKLR*, rs28928907 in *MPL*, rs33946267 in *HBB*, rs146220228 in *WAS*), or because the disease presented mild symptoms that are not easily detectable (rs35897051 and rs119468010 in *MPO* for myeloperoxidase deficiency).

We next compared the effects of PGS and rare monogenic variants. The average effect of each standard deviation of PGS ranged from 0.16 to 0.47 SD (depending on trait) and was thus comparable to that of a rare pathogenic variant carried in heterozygosity (Figure 6C). We hypothesized that in the previous examples, a low penetrance in a rare disease could be explained by background polygenic variation, for instance in cases where the rare disease mutation carriers have a polygenic effect in the opposite direction that compensated for a high-impact rare mutation. However, the PGS of identified homozygous rare variant carriers was not different from the population mean (defined arbitrarily as PGS > 2SD_PGS_ or tested by logistic regression for variants with more than 10 homozygotes). Hence, the polygenic effects alone were not sufficiently extreme to explain the lower disease prevalence in homozygous rare variant carriers, at least with our current PGS definition.

Comparing effect sizes can also be used to screen for potential new pathogenic mutations. Variants of uncertain significance (VUS) and missense variants showed a broad distribution of effect sizes, with tails approaching the range of pathogenic ones, and could harbor putative new pathogenic variants. Among the 16 missense variants with the largest effect sizes, two previously uncharacterized ones were in known Mendelian genes (rs139473150 in *TUBB1*, associated with platelet count and rs201514157 in *SPTA1* associated with immature reticulocytes). Platelet count was also associated with rs149254521 in *PEAR1,* a gene previously identified by an intronic variant in a platelet GWAS (Eicher et al., 2016). Subsequent functional studies showed that this gene is involved in platelet aggregation, which is consistent with the phenotypes observed here (Eicher et al., 2016; Keramati et al., 2019). Two missense variants associated with monocyte count (rs140221307 and rs149771513) were in *IL17RA* (CHARGE Consortium Hematology Working Group, 2016; Tajuddin et al., 2016), which has been implicated in monocyte function in mice (Ge et al., 2014). Similarly, *E2F4* is known to be essential in mouse erythropoiesis (Humbert et al., 2000), and here its missense variant rs61735430 was strongly associated with mean reticulocyte volume. The other variants were in *TIE1* and *PLEKHO2* associated to platelets (rs140190628 and rs143331139 respectively), *IFRD2* (rs200622087) associated with reticulocytes, *TF* (rs8177318 and rs150854910) with mean corpuscular hemoglobin (MCH) and MCV, *CXCR2* (rs61733609 and rs55799208) with white blood cells (Auer et al., 2014), *C10orf54* (rs201859625, in *VSIR* encoding V-set immunoregulatory receptor) with monocytes, and *FAM46C* (rs148397151) with MCH. The high predictive value of the PGS, alongside future catalogs of additional rare and private variants from whole genome sequencing (WGS), will enable robust modeling and could help explain the heterogeneity in many monogenic blood disorders.

As shown earlier, polygenic contributions can yield effect sizes on blood traits comparable to or larger than that of known monogenic variants. We therefore sought to explore whether they affect predisposition to rare diseases of the blood. In UK Biobank, we extracted ICD10 codes for a total of 29,080 patients and controls, with 423 blood diseases. We then considered the subset of these participants that were excluded from the discovery GWAS and we estimated their weighted sentinel-based PGS (as detailed earlier). We then fit logistic regression models to test the associations of the PGS with rare disorders of the blood. For the first time, we showed that PGSs derived for blood parameters can influence risk for several rare blood disorders (Figure 6D). For instance, we showed that a higher PGS for red cell count was positively associated with incidence of secondary polycythemia, a disorder characterized by elevated hematocrit (OR = 1.55, 95% CI = 1.21–2.00, p = 6.5×10^−4^). A high PGS for MCH was protective for iron deficiency anemia (OR = 0.86, 95% CI = 0.79–0.94, p = 9.1×10^−4^). A high PGS for neutrophil count decreased the risk of aplastic anemia (OR = 0.74, 95% CI = 0.64–0.87, p = 2.9×10^−4^), which manifests as cytopenias due to depletion of hematopoietic stem cells and failure of blood cell production (Pascutti et al., 2016). Finally, the PLT PGS was negatively associated with thrombocytopenia or low PLT (OR = 0.83, 95% CI = 0.76–0.91, p = 3.8×10^−5^). We replicated findings for platelet related PGSs in an independent cohort of 1,199 BPD patients and 7,308 controls with WGS (Turro et al., 2020) (Figure 6D). We showed that an increased PGS for PLT resulted in a protective effect against BPD disorders, including thrombocytopenia (OR = 0.92, 95% CI = 0.86–0.98, p = 0.007). These results refine our understanding of rare blood disease heterogeneity and the contribution of the polygenic background of an individual to the manifestations of a rare disease known to be caused by high-impact pathogenic variants.

## Discussion

Hematopoiesis is a highly regulated hierarchical process. Genetic variation leading to alteration of blood cell counts can teach us fundamental lessons about this process and serve as a paradigm for studying complex trait genetic architecture (Bao et al., 2019; Tardaguila and Soranzo, 2019). Here we present a large set of GWAS association results for a set of traits that illuminate numerous aspects of hematopoiesis. The magnitude of this discovery set enabled unprecedented statistical power to explore current paradigms in complex trait genetics as well as build a bridge between GWAS in general population cohorts and existing knowledge of monogenic blood disorders.

While an omnigenic model has recently been proposed to explain complex disease or trait architecture (Boyle, Li and Pritchard, 2017), this framework has been met with some skepticism (Wray et al., 2018). Demonstrating the validity of the omnigenic model has relevance for disease gene discovery for differentiating genes with a potential to underpin pathological variation in human traits from those that control variation within healthy physiological ranges. Here we leveraged the knowledge accrued on the hematopoietic system to carry out a first empirical assessment of this model in the context of blood cell trait variation. By defining core genes as those that are found in Mendelian blood disorders and using a coexpression network, we describe properties consistent with this omnigenic model. Specifically, when compared to other GWAS-associated genes, Mendelian genes (1) were enriched among GWAS hits, (2) harbored variants with larger effect sizes, (3) had predominantly blood-specific effects, (4) were coregulated, (5) had central properties in the coexpression network, and (6) were enriched among trans-eQTL targets. Other observations were inconclusive or suggestive of a more continuous (infinitesimal) pattern of inheritance: (1) there was an apparent continuum of effect sizes of variants on disease odds, (2) there was no significant difference in functional scores (chromatin accessibility, deltaSVM) between variants assigned to “core” versus “peripheral” genes, and (3) first- and second-degree neighbors of Mendelian genes were compatible with “core” and “peripheral” functions. The relatively incomplete ascertainment of rare and private DNA sequence variation in both population cohorts and rare disease cases implies that our knowledge of core genes is likely incomplete. Despite these uncertainties, the emerging picture of underlying network connectivity regulating blood traits harbors potential for discovering new pathogenic genes and drug targets. As an example, we identified a subset of 11 closely coexpressed genes (including three known platelet genes (*GP9*, *ITGA2B*, and *GP1BB*) that is coregulated by the same trans-acting eQTL in the *ARHGEF3* gene. The use of a large dataset with concurrent genetic and gene expression data in different cell states will be necessary for further quantitative validation of this model (Liu et al., 2019).

Polygenic variation has a substantial contribution to variation in complex quantitative traits and disease risk, sometimes yielding effects comparable to those of rare pathogenic variants. Using only the sentinel signals from our discovery GWAS, we built PGSs explaining up to 28% of phenotypic variance. We explored the polygenic effects jointly with pathogenic variants and as phenotype modulators in patients with rare blood disorders. While we found that 16 known monogenic variants were each associated with quantitative blood traits, 52 participants homozygous for five rare recessive pathogenic variants appeared to be healthy with normal blood count and indices. This suggests that the penetrance of pathogenic variants may be overestimated in many instances, as was recently shown (Oetjens et al., 2019). Differences in PGS could not explain the reduced penetrance, but our analysis may be limited by the diseases we had adequate statistical power to assess. Conversely, we observed strong allele dosage-dependent effect sizes for two heterozygous variants (previously reported as recessive), that could lead to disease especially if coinherited with an adverse PGS. For example, heterozygous carriers of the *GP9* variant rs5030764 were three times more likely to have a PLT below the normal range (< 150K/ul). Finally, we observed a significant association between phenotype-relevant PGSs and rare blood disorders for thrombocytopenia, secondary polycythemia, anemia, and aplastic anemia, regardless of the presence or absence of known rare variants in patients. This highlights a substantial polygenic modulating effect on presumably monogenic disorders and lays the groundwork for future studies aiming to define the impact of genetic background on the variable penetrance and expressivity in blood disorders.

In summary, through the largest study of blood cell trait variation to date, we provide new insights into the regulation of blood cell parameters and how genetic variation may contribute to the variability observed in rare blood disorders that are presumed to have a monogenic etiology. Our findings provide a novel framework for considering an individual’s genetic background and how this may impact the presentation of blood diseases. Finally, the lessons learned from this study of hematopoiesis will likely be more broadly applicable to a wide range of other complex diseases and traits.

## STAR★Methods

### Key Resources Table

**Table:** 

REAGENT or RESOURCE	SOURCE	IDENTIFIER
**Deposited Data**		

The NIHR BioResource, on behalf of the 100,000 Genomes Project	The NIHR BioResource, on behalf of the 100,000 Genomes Project (2019). Whole-genome sequencing of rare disease patients in a national healthcare system (bioRxiv)	https://www.genomicsengland.co.uk/about-gecip/joining-researchcommunity/
Ensembl v99	EMBL-EBI	https://www.ensembl.org/index.html;
HOCOMOCO	(Kulakovskiy et al., 2018)	https://repository.kaust.edu.sa/handle/10754/325453
PheWAS summary statistics from UK Biobank	Lee Lab, Seoul National University	https://www.leelabsg.org/resources
Geuvadis	(Lappalainen et al., 2013)	http://www.geuvadis.org
ClinVar database	https://www.ncbi.nlm.nih.gov/clinvar/	accessed 2018-11-05
Human Gene Mutation Database (HGMD)	http://www.hgmd.cf.ac.uk/ac/index.php	accessed 2019-01-17
eQTL-gen	University of Groningen	https://www.eqtlgen.org/
BIOS	BIOS Consortium	http://bbmri.researchlumc.nl/atlas/#data
ATAC-seq profiles	Gene Expression Omnibus (GEO) and Sequence Read Archive (SRA)	Accession GSE119453 (GEO) and PRJNA491478 (SRA)
UK Biobank summary statistics	This manuscipt	GWAS Catalog:GCST90002379-GCST90002407

**Software and Algorithms**		

R 3.6.1	R Core Team	https://www.r-project.org/
BOLT-LMM	(Loh et al. 2015)	https://alkesgroup.broadinstitute.org/BOLT-LMM/
checkVCF	Abecasis Lab - University of Michigan	https://genome.sph.umich.edu/wiki/CheckVCF.py
Sanger Imputation service	Wellcome Sanger Institute	https://imputation.sanger.ac.uk/
Michigan Imputation Server	University of Michigan	http://imputationserver.sph.umich.edu/index.html
EPACTS	Hyun Min Kang (University of Michigan)	https://github.com/statgen/EPACTS
rvtests	(Zhan et al., 2016)	https://github.com/zhanxw/rvtests
GWAMA	(Mägi and Morris, 2010)	https://www.well.ox.ac.uk/GWAMA
GCTA	(Yang et al., 2011)	https://gump.qimr.edu.au/gcta
FINEMAP v1.3.1	(Benner et al., 2016)	http://www.christianbenner.com/
gwas-pw	(Pickrell et al., 2016)	https://github.com/joepickrell/gwas-pw
g-chromVAR	(Ulirsch et al., 2019)	https://caleblareau.github.io/gchromVAR
DeltaSVM	(Lee et al., 2015)	http://www.beerlab.org/deltasvm/
SpliceAI	(Jaganathan, et al., 2019)	https://github.com/Illumina/SpliceAI.
ggsashimi	(Garrido-Martín et al., 2018)	https://github.com/guigolab/ggsashimi
PLINK v1.9	PLINK working group	https://www.cog-genomics.org/plink/1.9/
VEP: Variant Effect Predictor	EMBL-EBI	https://www.ensembl.org/Tools/VEP
LeafCutter	(Li et al., 2017)	https://davidaknowles.github.io/leafcutter/

### Resource Availability

#### Lead Contact

Further information and requests may be directed to the Lead Contact, Nicole Soranzo (ns6@sanger.ac.uk).

#### Materials Availability

This study did not generate new unique reagents.

#### Data and Code Availability

Summary statistics are available to download from: ftp://ftp.sanger.ac.uk/pub/project/humgen/summary_statistics/UKBB_blood_cell_traits/ for UK Biobank and http://www.mhi-humangenetics.org/en/resources for the meta-analysis. The accession numbers for the UK Biobank summary statistics reported in this paper are GWAS Catalog: GCST90002379–GCST90002407.

The code generated during this study is publicly available at GitHub https://github.com/bloodcellgwas/manuscript_code/.

### Experimental Model and Subject Details

Following the success of the Blood Cell Consortium ((Chami et al., 2016; Eicher et al., 2016) and (Tajuddin et al., 2016)), the Blood Cell Consortium Phase 2 (BCX2) continues to identify novel common and rare variants associated with blood cell traits using imputed genotype data based on Haplotype Reference Consortium (HRC) or the 1000 Genomes Project (Phase 3, version 5) for European ancestry cohorts and non-European ancestry cohorts, respectively. BCX2 comprises 746,667 participants from 40 discovery cohorts and five ancestries: European, African American, Hispanic, East Asian, and South Asian. BCX2 is divided into two working groups: European that consists of 563,946 participants from 26 cohorts and that is the focus of this study, and trans-ethnic. Detailed descriptions of the participating cohorts are summarized in Table S1. All participants provided written informed consent, and local research ethics committees and institutional review boards approved the individual studies.

### Method Details

#### Genotyping, quality control and imputation

Genotyping array and pre-imputation quality control (QC) for each participating cohort is provided in Table S2. Genotype QC metrics included MAF (> 0), call rate (> 98%) and Hardy-Weinberg equilibrium p (> 10^−6^). The pre-imputation sample exclusion criteria (Table S2) included call rate (> 95%), heterozygosity rate (> median+3^∗^IQR), gender mismatches, duplicates, and outliers from principal component analysis with reference samples from 1000 Genomes Project. All genotypes were on Genome Reference Consortium Human Build 37 (GRCh37) forward strand (https://www.well.ox.ac.uk/∼wrayner/strand/). All the cohorts checked strand and allele orientation in the variant call format files prior to imputation using checkVCF (https://genome.sph.umich.edu/wiki/CheckVCF.py). Finally the imputation was performed using servers available at https://imputation.sanger.ac.uk/ or http://imputationserver.sph.umich.edu/index.html with requesting the HRCr1.1 2016 reference panel, EUR population and Quality Control & Imputation Mode. All cohorts followed this procedure for the imputation of autosomal variants except for UK Biobank (UKBB) and INTERVAL that had their genotype imputation described elsewhere (Astle et al., 2016).

### Quantification and Statistical Analysis

#### Phenotype modeling and cohort level GWAS

When possible, we excluded samples with any of the following: pregnancy (when complete blood count (CBC) done), acute medical/surgical illness (when CBC done), blood cancer, leukemia, lymphoma, chemotherapy, myelodysplastic syndrome, bone marrow transplant, congenital or hereditary anemia (e.g., hemoglobinopathy such as sickle cell anemia or thalassemia), HIV, end-stage kidney disease, dialysis, EPO treatment, splenectomy, cirrhosis and those with any of the following extreme measurements: WBC count > 100^∗^10^9^/L with > 5% immature cell or blasts, WBC > 200^∗^10^9^/L, Hemoglobin > 20 g/dL, Hematocrit > 60%, Platelet > 1000^∗^10^9^/L. For the WBC subtypes (e.g., basophils count) we used the relative count, i.e., the total WBC count multiplied by the proportion for each cell type (e.g., basophils percentage). Raw phenotypes were regressed on age, age-squared, sex, principal components and cohort specific covariates (e.g., study center, cohort, etc) if needed, WBC related traits were log_10_ transformed before regression modeling. Residuals from the modeling were obtained and then inverse normalized for cohort level association analysis or GWAS. All cohorts followed the same exclusions and phenotype modeling except for UKBB and INTERVAL that had their procedure described elsewhere (Astle et al., 2016). The cohort level association analyses were then conducted using a linear mixed effects model in order to account for known or cryptic relatedness (e.g., BOLT-LMM (Loh et al., 2015, 2018), EPACTS https://github.com/statgen/EPACTS and rvtests (Zhan et al., 2016) with the additive genetic model. Linear mixed effects models have been shown to effectively account for both population structure and inter-individual relatedness within the UK Biobank cohort, along with having increased discovery power over simple linear regression with principal components.

#### QC and pre-processing of cohort level GWAS

Cohort level association analysis results went through a standard QC procedure (Winkler et al., 2014; Zhan et al., 2016) using EasyQC R package (https://www.uni-regensburg.de/medizin/epidemiologie-praeventivmedizin/genetische-epidemiologie/software/). The mapping file and allele frequency reference data (GRCh37/hg19) from HRC were used to harmonize variant names across cohorts and to check allele frequency discrepancies between cohorts and the HRC reference panel, respectively. We generated a unique ID for each variant using the form of chromosome:position_allele1_allele2 where alleles were ordered lexicographically or based on indel length as tri-allelic variants and/or indels of the same chromosome:position were observed. In addition to allele frequency plots, quantile-quantile (Q-Q) plots and SE-N (i.e., inverse of the median standard error versus square root of sample size) plots were also checked to detect systematic inflation, and different phenotypic variances due to mis-specified phenotype transformation or regression model, different study design or different study population, etc.

#### Meta-analysis

Post-QC’ed and pre-processed European cohort results were then meta-analyzed by GWAMA (Mägi and Morris, 2010) using inverse variance weighted fixed effects approach. We applied an imputation quality filter of INFO score ≤0.4 and a minor allele count (MAC) filter of MAC ≤5 for each variant in the meta-analysis, except for three large cohorts UKBB (N = 487,409), WHI (N = 17,682) and GERA (N = 53,822), where a more stringent MAC filter of MAC ≤ 20 was applied to exclude extremely rare variants with extreme effects prior to meta-analysis.

#### Exact conditional analysis

We performed an exact conditional analysis using a stepwise multiple linear regression (Astle et al., 2016) approach in UKBB. Stepwise multiple linear regression aims to identify a parsimonious subset of variants which explain the significant associations identified by univariable GWAS. For each blood phenotype, the set of genome wide significant variants was partitioned into the largest number of blocks such that no pair of blocks are separated by fewer than 5Mb, subject to the restriction that no block contained more than 2,500 variants. Blocks were generated independently for each phenotype. For each phenotype and each block, we identified a parsimonious set of variants explaining the signal in that block using a stepwise conditional linear regression algorithm. Each iteration of the algorithm had two stages: i) addition of variants to the model and ii) removal of variants from the model. Convergence occurred when neither addition nor removal of any variant improved the model fit sufficiently for a t test p < 8.31x10^−9^. The variants in the model at convergence represent a parsimonious set for the block.

Let “*M*” represent the ‘current model,’ The algorithm is initialised with *M* as the empty model, containing just an intercept term, and develops with the following steps:

1. Update *M* by inserting the variant in the block with the lowest univariable association p value, into the model.

2. In turn for each variant^∗^ in the block not in *M*, compare *M* to the model generated by augmenting *M* with the variant using a t test. Record the p value from each comparison.

3. If the least p value recorded in step 2 is greater than 8.31x10^−9^ terminate the algorithm.

4. Update *M* by adding the variant with the least p value recorded in step 2.

5. In turn for each variant in *M*, compare *M* to the model generated by removing the variant from *M,* using a t test. Record the p values from each comparison.

6. If the greatest p value recorded in step 5 is smaller than 8.31x10^−9^ go to step 2.

7. Update *M* by removing the variant with the greatest p value recorded in step 5.

8. Go to step 5.

^∗^ When comparing *M* to *M* augmented by a variant, we are testing to see if this new variant represents a genetic signal independent of the variants in *M*. In the situation where a potential new variant is in high LD (r^2^ > 0.9) with a variant already in *M* we assume that the this variant cannot represent an independent signal and we do not proceed to calculate its P value.

All linear regression was performed using the fastLM from the R package RcppEigen.

For each phenotype, following identification of conditionally significant variants in each block, all conditionally significant variants within each chromosome were put into a single linear model and tested with the same multiple stepwise linear regression algorithm described above, but starting at step 5. The union across chromosomes of the resulting sets of variants is the ‘conditionally significant’ list of variants for the blood cell phenotype, also referred to as “sentinel variants” throughout the text.

#### Meta-analysis conditional analysis

Using conditional and joint analysis as implemented in GCTA (Yang et al., 2011) (https://gump.qimr.edu.au/gcta), we identified independent association results in the meta-analyses at p < 5x10^−9^. To define novel associations, we tested these variants using the same exact multivariate approach as above, in the UKBB, while conditioning on the variants identified by the previous step.

#### Replication

We checked replication in the Million Veteran Project Cohort (Gaziano et al., 2016), for chromosome 1 and three different traits, one per each major cell type (platelet counts, lymphocyte counts and red cell counts). The replication significance threshold was set to a nominal level (p < 0.05) with the same direction of effect.

#### Fine-mapping

Statistical fine-mapping was performed in the UKBB cohort, using FINEMAP v1.3.1 (Benner et al., 2016) (http://www.christianbenner.com/). Input windows were defined as +- 250 kb from a conditionally independent signal. In case of multiple sentinels generating overlapping windows these were merged together, resulting in window size ranging from 500kb to 1.38Mb. The number of conditionally independent signals in each window was used as prior knowledge for the maximum number of causative variants to be searched (–n-causal-snps option) and the prior standard deviation for effect sizes was set to 0.08 (–prior-std option). The LD structure was computed from the same samples included in the GWAS analysis. 95% credible sets were defined as minimal sets of variants jointly covering at least 95% of the posterior probability of including the true causative signals.

#### eQTL Colocalization

We performed colocalization using gwas-pw *(**Pickrell et al., 2016**)* between GWAS of 10 hematological traits and transcriptomic profiling of Platelets, CD4^+^, CD8^+^, CD14^+^, CD15^+^, and CD19^+^ cells. Where MPV, PDW, PLT#, and PCT were colocalized with eQTLs from Platelets, NEUT# and NEUT% were colocalized with eQTLs from CD15^+^ cells, LYMPH# and LYMPH% were colocalized with eQTLs from CD4^+^, CD8^+^, and CD19^+^ cells, and MONO# and MONO% were colocalized with eQTLs from CD14^+^ cells. Loci of colocalization were defined by the recombination regions identified by (Berisa and Pickrell, 2016). Colocalization was only performed if the locus contained a conditionally independent variant in LD r^2^ > 0.8 with the eQTL sentinel. Results were filtered to include only those with posterior probability for colocalization higher than 80% resulting in a set of colocalized loci which are considered ‘highly likely’ to be colocalized (Sun et al., 2018).

#### Mendelian genes

The list of Mendelian genes was retrieved from a manually curated list, compiled as part of the NIHR BioResource rare disease sequencing project (Turro et al., 2020). It includes genes causative for stem cell and myeloid disorders (SMDs, 206 genes), bleeding, thrombotic and platelet disorders (BPD, 104 genes) and bone-marrow failure syndromes (BMF 80 genes). We refer to Mendelian SNPs as those assigned by VEP (worst consequence) to one of the Mendelian genes. To test for differences in absolute effect sizes we matched for MAF between Mendelian and other SNPs using the R package “MatchIt” (Ho et al., 2007), separately for each functional annotation, with at least 10 variants per group. We then tested the absolute effect size distribution shift using the Wilcoxon test as implemented in R.

#### Co-expression network

A co-expression matrix computed from the whole blood of 2,168 participants was used (Nath et al., 2017). The matrix quantifies correlations between genes, replicated across 2 different cohorts. The edges between genes were defined by imposing variable hard cut-offs on co-expression coefficients, e.g., two genes are linked in the network if their co-expression is higher than the cut-off. The following cut-offs were used (0.05, 0.1, 0.2, …, 0.8) but the results did not generally depend on the specific cut-off (unless otherwise stated). The overlap enrichment between GWAS genes and network genes was computed by random permutations of gene sets, in particular we used the following steps:

1. Annotate GWAS associations by VEP - this gives us a gene annotation for 83% of GWAS variants, which we refer to as “GWAS genes”

2. Iterations:

- Randomly select a set of genes from the Ensembl v99 list of protein coding genes (using the R function “sample”). The size of the set matches the size of the test set

- Overlap the random set of genes with GWAS genes to calculate how many of the random genes are among the GWAS associations

- Repeat 10,000 times

3. Compare the observed overlap with the background distribution

The numbers of links per gene were compared between Mendelian genes and all other genes by Wilcoxon test. For co-expression among Mendelian genes, median absolute co-expression coefficients were computed for equal sized random draws of genes.

The enrichment of trans-eQTLs targeting Mendelian genes was computed similarly to the above comparison between effect sizes of Mendelian and other GWAS genes. First, we downloaded median gene expression levels in whole blood from one of the cohorts included in eQTLGen (Võsa et al., 2018), BIOS (http://bbmri.researchlumc.nl/atlas/#data). We rank-inverse normalized the median expression levels. Then, to account for differences in power detection due to higher expression levels of Mendelian genes and higher Z-scores of Mendelian-targeting trans-eQTLs, we used the R package “MatchIt” (Ho et al., 2007) to select a matched subset. This included trans-eQTLs targeting non-Mendelian genes with matched Mendelian expression levels and Z-scores for trans-eQTLs targeting Mendelian genes. We included the maximum possible number of trans-eQTL-gene pairs with these characteristics (N = 9,258). Finally, we compared the number of trans-eQTLs per gene in the two groups by Wilcoxon test.

The enrichment of GWAS genes among first and second degree neighbors to Mendelian genes was computed as follows: i) determine the list of neighboring genes based on the specific cut-off, ii) intersect with 1000 random permutations of gene sets of the same size as the GWAS list, iii) compare to the actual intersection. The second degree neighbors were defined as neighboring genes to all first-degree neighbors.

#### g-chromVAR

Bias-corrected enrichment of blood trait variants for chromatin accessibility of 18 hematopoietic populations was performed using g-chromVAR, whose methodology has been previously described in detail (Ulirsch et al., 2019). In brief, this method weights chromatin features by fine-mapped variant posterior probabilities and computes the enrichment for each cell type versus an empirical background matched for GC content and feature intensity. For chromatin feature input, we used a consensus peak set for all hematopoietic cell types with a uniform width of 500 bp centered at the summit. For variant input, we included all variants with fine-mapped PP_FM_ > 0.1%.

#### DeltaSVM

DeltaSVM is a machine learning model which uses sequence composition to predict cell type-specific open chromatin (Lee et al., 2015). It then uses this sequence vocabulary to predict the change in chromatin accessibility from each variant. We trained on two ATAC-Seq datasets: 1) 18 hematopoietic populations sorted from bone marrow, and 2) 8 stages of primary erythroid differentiation. For each dataset, we trained on strong ATAC peaks in the > 80th percentile of counts matrix from each cell type. Standard 5-fold cross-validation was used to calculate AUROC. We then scored each variant with a posterior probability of association greater than 0.001 for all populations to determine variants predicted to alter chromatin accessibility. Here, a positive deltaSVM score is interpreted as a prediction where the variant increases chromatin accessibility whereas a negative score would reduce chromatin accessibility.

#### Transcription factor motif analysis

Prediction of the effects of fine-mapped variants on transcription factor binding sites (TFBS) was performed by using the motifbreakR package and a collection of 426 human TFBS models (HOCOMOCO) (Kulakovskiy et al., 2018). For 115,609 fine-mapped variants with PP_FM_ > 0.1%, we applied the ‘information content’ scoring algorithm and used a p cutoff of 1 × 10^−4^ for TFBS matches; all other parameters were kept at default settings.

#### Phenome-wide association study

To identify associations between blood trait variants and clinical phenotypes, we conducted a phenome-wide association study (PheWAS) using summary statistics of 1,403 clinical phenotypes analyzed from the UK Biobank (https://www.leelabsg.org/resources). As input, we started with 574 rare variants with 0.00005 < MAF < 0.01 which were either conditionally independent lead signals or had fine-mapped PP_FM_ > 0.50. To avoid studying phenotypes with too few cases to capture these low allele frequencies, we only included phenotype-variant results for which the expected_case_minor_AC, calculated as 2 ^∗^ variant_MAF ^∗^ num_cases, was greater than 25. This resulted in the final inclusion of 529 clinical phenotypes (case numbers ranging from 1,236 - 77,977) across 456 variants which had pheWAS data. The Bonferroni-corrected significance threshold for pheWAS was calculated as 0.05 / 529 phenotypes = 9.45x10^−5^.

#### Splice variant analysis

To predict splice variants, we used SpliceAI, a deep neural network that accurately predicts splice junctions from genomic sequence (Jaganathan et al., 2019). We obtained prediction scores for all possible single nucleotide variants in the reference genome, which were released along with the SpliceAI tool, and extracted scores for all variants with fine-mapped PP_FM_ > 0.001 in one or more blood traits from the UK Biobank GWAS. We considered a variant to have a putative splicing consequence if it had a delta score > 0.2 for one or more splicing consequences (acceptor gain, acceptor loss, donor gain, donor loss); this threshold was shown to be enriched for splice variants and have high sensitivity.

Validation of SpliceAI predictions was performed using RNA-seq data on lymphoblastoid cell lines (LCL) of 465 participants from the Geuvadis project 23 (http://www.geuvadis.org). We aligned paired-end reads to the hg19 reference genome with STAR, allowing for novel splice junctions. To systematically evaluate predicted splice-altering variants, we processed junction files for all 465 samples using the LeafCutter workflow and evaluated changes in splicing clusters that overlapped the variant (Li et al., 2018). For each variant, read alignments were merged into two groups, all carriers versus all non-carriers, and visualized in the form of sashimi plots using the ggsashimi tool (Garrido-Martín et al., 2018). The threshold for the minimum number of reads supporting a junction to be drawn was 100 for rs8113779 and rs12898397, and reduced to 15 for rs139178017 given the low number of carriers.

#### Polygenic scores

The polygenic scores (PGSs) were computed as weighted sums of genotypes, weighted by their effect size on the phenotype (beta coefficient), using the PLINK score function. Beta coefficient estimates were computed in UK Biobank and PGS scores were tested in an independent cohort (INTERVAL). Positive effect alleles were included in order to get a positive contribution for each carried allele and consequently a positive correlation with the phenotype. The following SNP inclusion criteria were compared: a) all genome-wide SNPs after LD pruning with PLINK at 0.8 cut-off; b) LD-pruned SNPs with GWAS p < (0.05, 5x10^−4^, 5x10^−6^, 5x10^−8^); c) conditionally independent variants and fine-mapped variants with posterior probability > 0.5; d) conditionally independent variants. Resulting PGSs were then standardized and Pearson’s R coefficients of correlation between each PGS and its relevant trait were computed for comparison. The phenotypic variance explained was computed as R^2^. Validation in a further independent cohort of French-Canadians (European ancestry) called CARTaGENE was performed using the same protocol, for the best performing PGS (conditionally independent variants). A linear regression model between the adjusted phenotypes and the PGS, adjusted by sex, age and principal components, was used to determine the PGS’s effect sizes per SD.

#### Discovery saturation

To explore discovery saturation we chose 4 large GWAS analyses in cohorts of increasing sample size: INTERVAL (N∼35k), UK BiLEVE (N∼43,5k), UK Biobank 1st release (N∼83k) and UKBiobank full cohort (N∼400k). For all of these we had conditionally independent associations identified by the same method as described above. For each trait and cohort we determined the number of conditionally independent variants detected by GWAS; the number of genes identified by these variants (using VEP worst consequence annotation); the number of associated loci and we further subset the genes as Mendelian or others. Associated loci were defined based on LD-blocks computed in Pickrell et al. (Berisa and Pickrell, 2016) which had at least one conditionally independent signal. First, a linear projection of the first 3 data points was visually inspected to determine in the 4th data point fitted the expected. Then 4 different regression models were tested to determine which one best described the full dataset: i) y∼x; ii) y∼sqrt(x); iii) y∼sqrt(x)+x; (iv) y∼log(x). Here y represents the counts (number of variants/genes/loci associated) and x represents the cohort size. Similarly we computed the heritability explained by the set of variants identified by each cohort and searched for the best fitting model. The heritability was computed as R^2^ of the multivariate model including the relevant variants in the full UK Biobank cohort. The model fitting the Mendelian genes versus others was computed across all pooled phenotypes.

#### Allelic series and Disease Colocalization

We performed pairwise colocalization analysis between GWAS studies of 29 hematological parameters from the UK Biobank cohort and 18 different autoimmune and inflammatory related disorders. Our analysis was performed using summary statistics collected following GWAS of the respective studies. An inner merge was performed with variants tested for each hematological parameter and each respective disease risk GWAS. Colocalization analysis was then performed following the same protocol described above for eQTLs. Allelic series were defined as fine-mapping blocks including 2 or more associated sentinels.

#### Polygenic effects in rare blood disorders

We used UK Biobank participants with ICD10 codes in Chapter III (“Diseases of the blood and blood-forming organs,” D500-D899 codes), who were excluded from the GWAS discovery. After computing the PGS, we performed a logistic regression for the disease status, including sex, age, 10 principal components and any other co-occurring blood disorders as covariates. P values were corrected by Bonferroni correction for the number of diseases (i.e., ICD10 codes) tested. We included only ICD10 codes with at least 40 cases for a total of 49 disorders.

#### Pathogenic variants annotation

Conditionally independent variants for each trait, as well as fine-mapped variants with posterior probability of being causative greater than 50% were pulled together for the pathogenicity annotation. Genes were assigned to variants by VEP worst consequence [release 84]. The set of Mendelian genes was manually curated by the NIHR-RD project. We focused on three different sources of pathogenicity annotations: the ClinVar database (https://www.ncbi.nlm.nih.gov/clinvar/ - accessed 2018-11-05), the Human Gene Mutation Database, version pro 2018.4 (HGMD) (http://www.hgmd.cf.ac.uk/ac/index.php - accessed 2019-01-17) and a manually curated list of novel pathogenic variants, produced by the NIHR-RD sequencing project (Turro et al., 2020). Variants were matched by chromosome, position and alleles, in GRCh37. The following parameters were considered: a) ClinVar: categorical pathogenicity assignment (yes/no/unknown), the star rating (1-4, 1 being the most uncertain and 4 the most certain) and the Review Status; b) HGMD: categorial pathogenicity assignment and Rank Score indicating the pathogenicity confidence on a continuous scale from 0 to 1, 1 being certain pathogenicity assignment and 0 being very uncertain. The set of pathogenic variants was defined with high confidence, imposing pathogenicity in ClinVar with at least 2 stars or pathogenicity in HGMD (“DM”) with rank score greater than 0.1. Variants reported by NIHR-RD for the first time were assigned to the “variants of uncertain significance” category (VUS). To assess the effects of such pathogenic variants and their penetrance, two types of data were considered: full blood count diagnostic cut-offs as used in the clinics and ICD-10 codes for blood disorders (Chapter III), as recorded by UKBB. Participants with full blood counts in the normal range and no ICD-10 code were considered healthy. The joint modeling of rare variants and PGS was performed only for variants with more than 10 homozygotes, using logistic regression and relevant covariates, as above. For variants with less than 10 homozygotes, we checked if these for systematic PGS deviation from the population mean (defined as PGS > 2^∗^SD_PGS_).

## Supplemental Information

Table S1. Phenotypes’ Description, Related to Figure 1 Summary of measurement methods for full blood count phenotypes used in discovery GWAS.

Table S2. BCX Consortium Cohort Information, Related to Figure 1 The study design, number of participants and sample quality control for all study cohorts is reported together with genotyping methods, SNP quality control criteria, imputation, and statistical analysis used.

Table S3. Conditionally Independent Associations in UK Biobank, Related to Figure 1 Information about each of the 17,042 variants associated and their corresponding sentinel variants is given, ordered by chromosome and position (all coordinates are with respect to GRCh37). Locus ID is a unique identifier for each locus. The column “Novel vs Astle et al. 2016” indicates if the variant or any variants in LD r^2^>0.8 was already found to be associated with the same traits in the cited previously published meta-analysis including the first release of UK Biobank. The unique variant ID is constructed from the chromosome, position and the reference and alternative alleles according to the human genome reference (build 37 coordinates). Where available, the rsID is also given. GWAS summary statistics for univariate and multivariate (conditional) model are provided, as well as the VEP worst consequence annotation.

Table S4. Additional Conditionally Independent Associations from the Meta-analysis, Related to Figure 1 Variants identified using GCTA-COJO that are genome-wide significant (p < 5e-9) and conditionally independent from the UKBB-only association results. All joint results were calculated after regressing out the effect of the variants found in the UKBB-only analyses.

Table S5. Fine-Mapping Posterior Probabilities, Related to Figure 1 The table reports all the sentinels fine-mapped to a single causative signal, i.e., having FM_PP_ > 0.95.

Table S6. Network Connectivity by Annotation, Related to Figure 2

Table S7. NIHR-RD List of Mendelian Genes, Related to Figure 2 The table shows a manually curated list of 314 genes known to cause blood disorders. The list was compiled by expert clinicians involved in the NIHR-RD sequencing project and is to date the most updated resource for blood disease genes and mutations. The genes are divided in 3 (possibly overlapping) categories: Stem cell and Myeloid Disorders (SMD), Bleeding, Thrombotic and Platelet Disorders (BPD) and Bone Marrow Failure (BMF).

Table S8. PheWAS Results, Related to Figure 4 Phenome-wide association study (pheWAS) significant associations between blood trait variants and other clinical phenotypes. var, variant ID in chromosome:position:allele1:allele2 format; ID, variant RSID; pheno, pheWAS phenotype associated with variant; trait, blood trait associated with variant; effect_allele, allele corresponding to GWAS effect sizes; blood_beta; GWAS effect size for the blood phenotype; PP_FM_, fine-mapped posterior probability; VEP_cons_most_serious, VEP most serious consequence; num_cases, number of cases of the pheWAS phenotype; maf, variant minor allele frequency; beta, GWAS effect size for the pheWAS phenotype; sebeta, GWAS standard error of the effect size for the pheWAS phenotype; pval, GWAS p value for the pheWAS phenotype; nearest_gene, nearest gene to the variant.

Table S9. Splice Variants Associated with Blood Traits, Related to Figure 4 Putative splice variants fine-mapped for blood trait associations (SpliceAI delta score > 0.2 and fine-mapped PP_FM_ > 0.001). var, variant ID in chromosome:position:allele1:allele2 format; rsid, variant RSID; chr, chromosome; pos, position; ref, reference allele; alt, alternate allele; SYMBOL, spliced target gene; STRAND, strand with splicing effect; DIST, distance between canonical and newly created splice sites; DS_AG, Delta score (acceptor gain); DS_AL, Delta score (acceptor loss); DS_DG, Delta score (donor gain); DS_DL, Delta score (donor loss); DP_AG, Delta score (acceptor gain); DP_AL, Delta score (acceptor loss); DP_DG, Delta score (donor gain); DP_DL, Delta score (donor loss); PP_FM_, fine-mapped posterior probability; AF_Allele2, frequency of alternate allele; trait, blood trait; frame, effect of splice variant on reading frame.

Table S10. PGS Correlation, Related to Figure 5 Results for all tested polygenic scores are shown as the R (Pearson correlation coefficient) between the score and the real phenotype in the INTERVAL cohort. For each set of SNPs, the PGS was tested in the entire cohort and in males only (M) to ensure that no sex bias was present. Column Q shows the R^2^ (i.e., the trait variance explained) for the best score, which includes conditionally independent variants only. Abbreviations: GW = genome-wide.

Document S1. Extended Acknowledgments and Author Contributions
