# Supplementary material for: The Polygenic and Monogenic Basis of Blood Traits and Diseases
Source: Cell. 2020 Sep 3;182(5):1214–1231.e11. doi: 10.1016/j.cell.2020.08.008 (PMC7482360; doi:10.1016/j.cell.2020.08.008)
Supplement: Table S6. Network Connectivity by Annotation, Related to Figure 2 [file mmc6.pdf]

Coexpression cut-off	N. genes in network	N. network genes covered by gwas (VEP) with intergenic	% of total In network	N. network genes covered by ALL VEP conseq	% of total In network	N. network genes covered by 250kb window (equivalent to FM Block)	% of total In network	N. mendelian genes in network
0	7509	1874	24.9567186	2070	27.5669197	5720	76.17525636	134
0.1	7509	1874	24.9567186	2070	27.5669197	5720	76.17525636	134
0.2	7430	1866	25.11440108	2061	27.73889637	5673	76.3526245	132
0.3	6946	1777	25.58306939	1968	28.33285344	5350	77.0227469	131
0.4	5994	1578	26.32632633	1745	29.11244578	4668	77.87787788	114
0.5	4554	1220	26.78963549	1339	29.40272288	3551	77.97540624	92
0.6	2657	726	27.32404968	794	29.88332706	2071	77.94505081	62
0.7	1051	300	28.54424358	330	31.39866794	802	76.30827783	38
0.8	262	95	36.25954198	102	38.93129771	202	77.09923664	15
0.9	20	8	40	8	40	19	95	2

Supplementary Table 6. Related to Figure 2; Network connectivity by annotation.
The co-expression network characteristics are shown based on different correlation coefficient cut-offs applied. The correlation cut-off identifies genes that are co-expressed with at least one other gene and an absolute correlation higher than the cut-off. Hence higher cut-off values identify smaller networks (columns A and B). The remaining columns show the overlap between the network and the genes identified by GWAS, based on different annotation strategies (absolute numbers and percentages are shown). Finally, column I shows how many Mendelian genes are included in the relevant network.
