# Supplementary material for: The Polygenic and Monogenic Basis of Blood Traits and Diseases
Source: Cell. 2020 Sep 3;182(5):1214–1231.e11. doi: 10.1016/j.cell.2020.08.008 (PMC7482360; doi:10.1016/j.cell.2020.08.008)
Supplement: Document S1. Extended Acknowledgments and Author Contributions [file mmc11.docx]

**EXTENDED ACKNOWLEDGMENTS AND AUTHOR CONTRIBUTIONS**

**ACKNOWLEDGMENTS**

Participants in the INTERVAL randomised controlled trial were recruited with the active collaboration of NHS Blood and Transplant England (www.nhsbt.nhs.uk), which has supported field work and other elements of the trial. DNA extraction and genotyping was co-funded by the National Institute for Health Research (NIHR), the NIHR BioResource (http://bioresource.nihr.ac.uk) and the NIHR [Cambridge Biomedical Research Centre at the Cambridge University Hospitals NHS Foundation Trust] [*]. The academic coordinating centre for INTERVAL was supported by core funding from: NIHR Blood and Transplant Research Unit in Donor Health and Genomics (NIHR BTRU-2014-10024), UK Medical Research Council (MR/L003120/1), British Heart Foundation (SP/09/002; RG/13/13/30194; RG/18/13/33946) and the NIHR [Cambridge Biomedical Research Centre at the Cambridge University Hospitals NHS Foundation Trust] [*]. A complete list of the investigators and contributors to the INTERVAL trial is provided in reference [**]. The academic coordinating centre would like to thank blood donor centre staff and blood donors for participating in the INTERVAL trial.

This work was supported by Health Data Research UK, which is funded by the UK Medical Research Council, Engineering and Physical Sciences Research Council, Economic and Social Research Council, Department of Health and Social Care (England), Chief Scientist Office of the Scottish Government Health and Social Care Directorates, Health and Social Care Research and Development Division (Welsh Government), Public Health Agency (Northern Ireland), British Heart Foundation and Wellcome.

*The views expressed are those of the authors and not necessarily those of the NHS, the NIHR or the Department of Health and Social Care.

**Di Angelantonio E, Thompson SG, Kaptoge SK, Moore C, Walker M, Armitage J, Ouwehand WH, Roberts DJ, Danesh J, INTERVAL Trial Group. Efficiency and safety of varying the frequency of whole blood donation (INTERVAL): a randomised trial of 45 000 donors. Lancet. 2017 Nov 25;390(10110):2360-2371.

This research has been conducted using the UK Biobank Resource under Application Number 13745.

This research was supported by NHLBI Intramural Research Funding to ADJ. The views expressed in this manuscript are those of the authors and do not necessarily represent the views of the National Heart, Lung, and Blood Institute, the NIH, or the U.S. Department of Health and Human Services. The NHLBI’s Framingham Heart Study is a joint project of the National Institutes of Health and Boston University School of Medicine and was supported by contract N01-HC-25195.

**Disclaimer**: Where authors are identified as personnel of the International Agency for Research on Cancer / World Health Organization, the authors alone are responsible for the views expressed in this article and they do not necessarily represent the decisions, policy or views of the International Agency for Research on Cancer / World Health Organization.

| **First** | **Last** | **Contribution** | **Declaration of Interests** | **Acknowledgments** |
| --- | --- | --- | --- | --- |
| Parsa | Akbari | Conceptualization, Methodology, Software, Formal Analysis, Writing – Original Draft |  | PA is funded by the National Institute for Health Research Blood and Transplant Research Unit in Donor Health and Genomics (NIHR BTRU-2014-10024). |
| Masato | Akiyama | Conceptualization, Resources |  |  |
| Patrick | Albers | Resources, Data Curation, Writing – Review & Editing |  |  |
| William | Astle | Conceptualization, Resources, Supervision, Writing – Original Draft |  | WJA is funded by the National Institute for Health Research [Cambridge Biomedical Research Centre at the Cambridge University Hospitals NHS Foundation Trust] [*]. *The views expressed are those of the authors and not necessarily those of the NHS, the NIHR or the Department of Health and Social Care. |
| Paul | Auer | Conceptualization, Resources, Supervision, Writing – Original Draft |  | 1R01HL130733-01A1 |
| Erik | Bao | Conceptualization, Methodology, Software, Formal Analysis, Writing – Original Draft |  | ELB received support from the Howard Hughes Medical Institute Medical Research Fellowship |
| Traci | Bartz | Conceptualization, Resources |  |  |
| Yoav | Ben-Shlomo | Conceptualization, Resources |  | The Caerphilly Prospective Study was undertaken by the former MRC Epidemiology Unit (South Wales) and was funded by the Medical Research Council of the United Kingdom. The Caerphilly DNA bank was established by an MRC Grant (G9824960). |
| Andrew | Beswick | Conceptualization, Resources |  |  |
| Jette | Bork-Jensen | Conceptualization, Resources |  | Novo Nordisk Foundation Center for Basic Metabolic Research is an independent Research Center, based at the University of Copenhagen, Denmark and partially funded by an unconditional donation from the Novo Nordisk Foundation (www.cbmr.ku.dk [cbmr.ku.dk]) (Grant number NNF18CC0034900). |
| Erwin | Bottinger | Conceptualization, Resources |  |  |
| Jennifer | Brody | Conceptualization, Resources |  |  |
| Linda | Broer | Conceptualization, Resources |  | The Rotterdam Study is funded by Erasmus Medical Center and Erasmus University, Rotterdam, Netherlands Organization for the Health Research and Development (ZonMw), the Research Institute for Diseases in the Elderly (RIDE), the Ministry of Education, Culture and Science, the Ministry for Health, Welfare and Sports, the European Commission (DG XII), and the Municipality of Rotterdam. The authors are grateful to the study participants, the staff from the Rotterdam Study and the participating general practitioners and pharmacists. |
| Adam | Butterworth | Conceptualization, Resources, Supervision, Writing – Original Draft | ASB has received grants (outside of this work) from AstraZeneca, Biogen, BioMarin, Bioverativ, Merck, Novartis and Sanofi |  |
| Na | Cai | Conceptualization, Resources |  |  |
| Ming-Huei | Chen | Conceptualization, Methodology, Software, Formal Analysis, Writing – Review & Editing |  |  |
| Kumaraswamy | Chitrala | Conceptualization, Resources |  |  |
| Kelly | Cho | Conceptualization, Resources |  |  |
| Hélène | Choquet | Conceptualization, Resources |  | Genotyping of the GERA cohort was funded by a grant from the National Institute on Aging, National Institute of Mental Health, and National Institute of Health Common Fund (RC2 AG036607). E.J. and H.C. are supported by National Eye Institute (NEI) grant R01 EY027004 (E.J.) and National Institute of Diabetes and Digestive and Kidney Diseases grant R01 DK116738 (E.J.) |
| John | Danesh | Conceptualization, Supervision, Funding Acquisition |  | JD is a British Heart Foundation Professor, European Research Council Senior Investigator, and National Institute for Health Research (NIHR) Senior Investigator. JD is funded by the National Institute for Health Research [Senior Investigator Award] [*]. *The views expressed are those of the authors and not necessarily those of the NHS, the NIHR or the Department of Health and Social Care. |
| Emanuele | Di Angelantonio | Conceptualization, Supervision, Funding Acquisition |  |  |
| Niki | Dimou | Conceptualization, Resources |  |  |
| Jingzhong | Ding | Conceptualization, Resources |  |  |
| Paul | Elliott | Conceptualization, Resources |  | The Airwave Health Monitoring Study was funded by the UK Home Office,(2003-2019: grant number 780-TETRA) and is currently funded by the UK Medical Research Council and Economic and Social Research Council (ESRC) (MR/R023484/1) with additional support from the National Institute for Health Research Imperial College Biomedical Research Centre and Imperial College Health Care NHS Trust. P.E. acknowledges support from the Medical Research Council (MRC) Centre for Environment and Health (MR/L01341X/1, MR/S019669/1), the UK Dementia Research Institute (UKDRI) at Imperial College London, funded by the MRC, Alzheimer’s Society and Alzheimer’s Research UK; and Health Data Research UK (HDR-UK), funded by a consortium led by the UK Medical Research Council. We thank all participants in the Airwave Health Monitoring Study. This work used computing resources provided by the MRC- funded UK MEDical Bioinformatics partnership programme (UK MED-BIO) (MR/L01632X/1). |
| Tõnu | Esko | Conceptualization, Resources |  |  |
| Evangelos | Evangelou | Conceptualization, Resources |  |  |
| Michele | Evans | Conceptualization, Resources |  | The Healthy Aging in Neighborhoods of Diversity across the Life Span (HANDLS) study is funded by the intramural Research program of the National Institute on Aging, National Institutes of Health Grant AG-000513. |
| Stephan | Felix | Conceptualization, Resources |  | SHIP is part of the Community Medicine Research net of the University of Greifswald, Germany (www.community-medicine.de), which is funded by the Federal Ministry of Education and Research (grants no. 01ZZ9603, 01ZZ0103, and 01ZZ0403), the Siemens AG, the Ministry of Cultural Affairs as well as the Social Ministry of the Federal State of Mecklenburg-West Pomerania, and the network ‘Greifswald Approach to Individualized Medicine (GANI_MED)’ funded by the Federal Ministry of Education and Research (grant 03IS2061A). ExomeChip data have been supported by the Federal Ministry of Education and Research (grant no. 03Z1CN22) and the Federal State of Mecklenburg-West Pomerania. |
| James | Floyd | Conceptualization, Resources | Has consulted for Shionogi Inc |  |
| Michel | Georges | Conceptualization, Resources |  |  |
| Mohsen | Ghanbari | Conceptualization, Resources |  | The Rotterdam Study is funded by Erasmus Medical Center and Erasmus University, Rotterdam, Netherlands Organization for the Health Research and Development (ZonMw), the Research Institute for Diseases in the Elderly (RIDE), the Ministry of Education, Culture and Science, the Ministry for Health, Welfare and Sports, the European Commission (DG XII), and the Municipality of Rotterdam. The authors are grateful to the study participants, the staff from the Rotterdam Study and the participating general practitioners and pharmacists. |
| Niels | Grarup | Conceptualization, Resources |  | Novo Nordisk Foundation Center for Basic Metabolic Research is an independent Research Center, based at the University of Copenhagen, Denmark and partially funded by an unconditional donation from the Novo Nordisk Foundation (www.cbmr.ku.dk [cbmr.ku.dk]) (Grant number NNF18CC0034900). |
| Andreas | Greinacher | Conceptualization, Resources |  | SHIP is part of the Community Medicine Research net of the University of Greifswald, Germany (www.community-medicine.de), which is funded by the Federal Ministry of Education and Research (grants no. 01ZZ9603, 01ZZ0103, and 01ZZ0403), the Siemens AG, the Ministry of Cultural Affairs as well as the Social Ministry of the Federal State of Mecklenburg-West Pomerania, and the network ‘Greifswald Approach to Individualized Medicine (GANI_MED)’ funded by the Federal Ministry of Education and Research (grant 03IS2061A). ExomeChip data have been supported by the Federal Ministry of Education and Research (grant no. 03Z1CN22) and the Federal State of Mecklenburg-West Pomerania. |
| Michael | Guo | Conceptualization, Resources |  |  |
| Qi | Guo | Methodology, Software, Formal Analysis | Q.G. is a full-time employee of BenevolentAI |  |
| Jeff | Haessler | Conceptualization, Resources |  |  |
| Torben | Hansen | Conceptualization, Resources |  | Novo Nordisk Foundation Center for Basic Metabolic Research is an independent Research Center, based at the University of Copenhagen, Denmark and partially funded by an unconditional donation from the Novo Nordisk Foundation (www.cbmr.ku.dk [cbmr.ku.dk]) (Grant number NNF18CC0034900). |
| Joanna | Howson | Conceptualization, Resources | During the drafting of the manuscript JMMH became a fulltime employee of Novo Nordisk | JMMH was funded by British Heart Foundation (RG/13/13/30194; RG/18/13/33946) and funded by the National Institute for Health Research [Cambridge Biomedical Research Centre at the Cambridge University Hospitals NHS Foundation Trust] [*]. *The views expressed are those of the authors and not necessarily those of the NHS, the NIHR or the Department of Health and Social Care. |
| Wei | Huang | Conceptualization, Resources |  | We thank the National Institute for Nutrition and Health, Chinese Center for Disease Control and Prevention, the Chinese National Human Genome Center at Shanghai, the Carolina Population Center, the University of North Carolina at Chapel Hill, and all of the participants and study investigators involved in the China Health and Nutrition Survey. Data collection and analysis was supported by the Carolina Population Center (P2C HD050924, T32 HD007168), the NIH (R01HD30880, R01 DK056350, R24 HD050924, R01 HD38700, R01 DK072193 and U01 DK105561), the NIH Fogarty International Center (D43 TW009077, D43 TW007709), the China Ministry of Health, the Chinese National Human Genome Center at Shanghai, the China-Japan Friendship Hospital, and the Beijing Municipal Center for Disease Prevention and Control. |
| Jennifer | Huffman | Conceptualization, Methodology, Software, Formal Analysis, Writing – Review & Editing |  |  |
| Michael | Inouye | Conceptualization, Resources, Supervision, Writing – Review & Editing |  | MI is funded by the National Institute for Health Research [Cambridge Biomedical Research Centre at the Cambridge University Hospitals NHS Foundation Trust] [*]. *The views expressed are those of the authors and not necessarily those of the NHS, the NIHR or the Department of Health and Social Care. |
| Tao | Jiang | Conceptualization, Methodology, Software, Formal Analysis, Writing – Review & Editing |  | TJ is funded by the National Institute for Health Research [Cambridge Biomedical Research Centre at the Cambridge University Hospitals NHS Foundation Trust] [*]. *The views expressed are those of the authors and not necessarily those of the NHS, the NIHR or the Department of Health and Social Care. |
| Andrew | Johnson | Conceptualization, Resources, Supervision, Writing – Original Draft |  | This research was supported by NHLBI Intramural Research Funding to ADJ. The views expressed in this manuscript are those of the authors and do not necessarily represent the views of the National Heart, Lung, and Blood Institute, the NIH, or the U.S. Department of Health and Human Services. The NHLBI’s Framingham Heart Study is a joint project of the National Institutes of Health and Boston University School of Medicine and was supported by contract N01-HC-25195. |
| Eric | Jorgenson | Conceptualization, Resources |  | Genotyping of the GERA cohort was funded by a grant from the National Institute on Aging, National Institute of Mental Health, and National Institute of Health Common Fund (RC2 AG036607). E.J. and H.C. are supported by National Eye Institute (NEI) grant R01 EY027004 (E.J.) and National Institute of Diabetes and Digestive and Kidney Diseases grant R01 DK116738 (E.J.) |
| Tim | Kacprowski | Conceptualization, Resources |  | SHIP is part of the Community Medicine Research net of the University of Greifswald, Germany (www.community-medicine.de), which is funded by the Federal Ministry of Education and Research (grants no. 01ZZ9603, 01ZZ0103, and 01ZZ0403), the Siemens AG, the Ministry of Cultural Affairs as well as the Social Ministry of the Federal State of Mecklenburg-West Pomerania, and the network ‘Greifswald Approach to Individualized Medicine (GANI_MED)’ funded by the Federal Ministry of Education and Research (grant 03IS2061A). ExomeChip data have been supported by the Federal Ministry of Education and Research (grant no. 03Z1CN22) and the Federal State of Mecklenburg-West Pomerania. |
| Mika | Kähönen | Conceptualization, Resources |  | The Finnish Cardiovascular Study (FINCAVAS) has been financially supported by the Competitive Research Funding of the Tampere University Hospital (Grant 9M048 and 9N035), the Finnish Cultural Foundation, the Finnish Foundation for Cardiovascular Research, the Emil Aaltonen Foundation, Finland, the Tampere Tuberculosis Foundation, EU Horizon 2020 (grant 755320 for TAXINOMISIS; grant 848146 for To_Aition), and the Academy of Finland grant 322098. |
| Yoichiro | Kamatani | Conceptualization, Resources |  |  |
| Masahiro | Kanai | Conceptualization, Resources |  |  |
| Savita | Karthikeyan | Conceptualization, Resources |  | SK is funded by a BHF Programme Grant (RG/18/13/33946) |
| Julian | Knight | Conceptualization, Resources |  | JK is supported by a Wellcome Trust Investigator Award (204969/Z/16/Z) and the NIHR Oxford Biomedical Research  Centre. This work was funded by the Wellcome Trust (074318, 088891 and 090532/Z/09/Z), the European Research Council (ERC) under the European Union’s Seventh Framework Programme (281824) and the Medical Research Council (98082). |
| Fotis | Koskeridis | Conceptualization, Resources |  |  |
| Kousik | Kundu | Conceptualization, Resources |  |  |
| Leslie | Lange | Conceptualization, Resources |  |  |
| Caleb | Lareau | Conceptualization, Methodology, Software, Formal Analysis, Writing – Original Draft |  |  |
| Terho | Lehtimäki | Conceptualization, Resources |  | The Young Finns Study has been financially supported by the Academy of Finland: grants 322098, 286284, 134309 (Eye), 126925, 121584, 124282, 129378 (Salve), 117787 (Gendi), and 41071 (Skidi); the Social Insurance Institution of Finland; Competitive State Research Financing of the Expert Responsibility area of Kuopio, Tampere and Turku University Hospitals (grant X51001); Juho Vainio Foundation; Paavo Nurmi Foundation; Finnish Foundation for Cardiovascular Research ; Finnish Cultural Foundation; The Sigrid Juselius Foundation; Tampere Tuberculosis Foundation; Emil Aaltonen Foundation; Yrjö Jahnsson Foundation; Signe and Ane Gyllenberg Foundation; Diabetes Research Foundation of Finnish Diabetes Association; EU Horizon 2020 (grant 755320 for TAXINOMISIS; grant 848146 for To_Aition); European Research Council (grant 742927 for MULTIEPIGEN project); and Tampere University Hospital Supporting Foundation. |
| Guillaume | Lettre | Conceptualization, Resources, Supervision, Writing – Original Draft, Funding Acquisition |  | We thank all participants and staff of the André and France Desmarais MHI Biobank. This work was funded by the Canadian Institutes of Health Research (PJT #156248), the Canada Research Chair Program, Genome Quebec and Genome Canada, and the Montreal Heart Institute Foundation. |
| Yun | Li | Conceptualization, Resources |  | Yun Li is supported by funds of the NIH (R01 HL129132) |
| Allan | Linneberg | Conceptualization, Resources |  |  |
| Yongmei | Liu | Conceptualization, Resources |  |  |
| Ken Sin | Lo | Conceptualization, Resources |  |  |
| Ruth | Loos | Conceptualization, Resources |  | Ruth Loos is supported by funds of the NIH (R01DK110113; R01DK107786; R01HL142302; R01DK110113) |
| Leo-Pekka | Lyytikäinen | Conceptualization, Resources |  |  |
| Regina | Manansala | Conceptualization, Resources |  | 1R01HL130733-01A1 |
| Ani | Manichaikul | Conceptualization, Resources |  | Support for the statistical analyses in MESA was provided by R01 HL120393 and R01 HL105756. MESA and the MESA SHARe projects are conducted and supported by the National Heart, Lung, and Blood Institute (NHLBI) in collaboration with MESA investigators. Support for MESA is provided by contracts HHSN268201500003I, N01-HC-95159, N01-HC-95160, N01-HC-95161, N01-HC-95162, N01-HC-95163, N01-HC-95164, N01-HC-95165, N01-HC-95166, N01-HC-95167, N01-HC-95168, N01-HC-95169, UL1-TR-000040, UL1-TR-001079, UL1-TR-001420. Also supported in part by the National Center for Advancing Translational Sciences, CTSI grant UL1TR001881, and the National Institute of Diabetes and Digestive and Kidney Disease Diabetes Research Center (DRC) grant DK063491. Funding for SHARe genotyping was provided by NHLBI Contract N02-HL-64278. Genotyping was performed at Affymetrix (Santa Clara, California, USA) and the Broad Institute of Harvard and MIT (Boston, Massachusetts, USA) using the Affymetrix Genome-Wide Human SNP Array 6.0. |
| Koichi | Matsuda | Conceptualization, Resources |  |  |
| Karyn | Megy | Resources, Data Curation, Writing – Review & Editing |  | This study makes use of data generated by the NIHR BioResource. We thank NIHR BioResource volunteers for their participation, and gratefully acknowledge NIHR BioResource centres, NHS Trusts and staff for their contribution. We thank the National Institute for Health Research and NHS Blood and Transplant. The views expressed are those of the author(s) and not necessarily those of the NHS, the NIHR or the Department of Health and Social Care |
| Karen | Mohlke | Conceptualization, Resources |  |  |
| Nina | Mononen | Conceptualization, Resources |  |  |
| Arden | Moscati | Conceptualization, Resources |  |  |
| Abdou | Mousas | Conceptualization, Methodology, Software, Formal Analysis, Writing – Review & Editing |  |  |
| Yoshinori | Murakami | Conceptualization, Resources |  |  |
| Girish | Nadkarni | Conceptualization, Resources |  |  |
| Kjell | Nikus | Conceptualization, Resources |  |  |
| Yukinori | Okada | Conceptualization, Resources |  | Y.O. was supported by the Japan Society for the Promotion of Science (JSPS) KAKENHI (15H05911, 19H01021), AMED (JP19gm6010001, JP19ek0410041, JP19ek0109413, and JP19km0405211), and Takeda Science Foundation. |
| Willem | Ouwehand | Conceptualization, Resources, Supervision, Writing – Original Draft |  | W.H.O. is a NIHR Senior Investigator |
| Nathan | Pankratz | Conceptualization, Resources |  |  |
| Oluf | Pedersen | Conceptualization, Resources |  | Novo Nordisk Foundation Center for Basic Metabolic Research is an independent Research Center, based at the University of Copenhagen, Denmark and partially funded by an unconditional donation from the Novo Nordisk Foundation (www.cbmr.ku.dk [cbmr.ku.dk]) (Grant number NNF18CC0034900). |
| Christopher | Penkett | Resources, Data Curation, Writing – Review & Editing |  |  |
| Hannes | Ponstingl | Resources, Data Curation, Writing – Review & Editing |  |  |
| Michael | Preuss | Conceptualization, Resources |  |  |
| Bruce | Psaty | Conceptualization, Resources |  | Cardiovascular Health Study: This CHS research was supported by NHLBI contracts HHSN268201200036C, HHSN268200800007C, HHSN268201800001C, N01HC55222, N01HC85079, N01HC85080, N01HC85081, N01HC85082, N01HC85083, N01HC85086,R01HL068986; and NHLBI grants U01HL080295, R01HL087652, R01HL105756, R01HL103612, R01HL120393, and U01HL130114 with additional contribution from the National Institute of Neurological Disorders and Stroke (NINDS). Additional support was provided through R01AG023629 from the National Institute on Aging (NIA). A full list of principal CHS investigators and institutions can be found at CHS-NHLBI.org [chs-nhlbi.org]. The provision of genotyping data was supported in part by the National Center for Advancing Translational Sciences, CTSI grant UL1TR001881, and the National Institute of Diabetes and Digestive and Kidney Disease Diabetes Research Center (DRC) grant DK063491 to the Southern California Diabetes Endocrinology Research Center. The content is solely the responsibility of the authors and does not necessarily represent the official views of the National Institutes of Health. |
| Huijun | Qian | Conceptualization, Resources |  |  |
| Laura | Raffield | Conceptualization, Methodology, Software, Formal Analysis, Writing – Review & Editing |  | T32 HL129982 |
| Olli | Raitakari | Conceptualization, Resources |  | The Young Finns Study has been financially supported by the Academy of Finland: grants 322098, 286284, 134309 (Eye), 126925, 121584, 124282, 129378 (Salve), 117787 (Gendi), and 41071 (Skidi); the Social Insurance Institution of Finland; Competitive State Research Financing of the Expert Responsibility area of Kuopio, Tampere and Turku University Hospitals (grant X51001); Juho Vainio Foundation; Paavo Nurmi Foundation; Finnish Foundation for Cardiovascular Research ; Finnish Cultural Foundation; The Sigrid Juselius Foundation; Tampere Tuberculosis Foundation; Emil Aaltonen Foundation; Yrjö Jahnsson Foundation; Signe and Ane Gyllenberg Foundation; Diabetes Research Foundation of Finnish Diabetes Association; EU Horizon 2020 (grant 755320 for TAXINOMISIS; grant 848146 for To_Aition); European Research Council (grant 742927 for MULTIEPIGEN project); and Tampere University Hospital Supporting Foundation. |
| Alexander | Reiner | Conceptualization, Resources, Supervision, Writing – Original Draft |  | R01 HL129132 |
| Stephen | Rich | Conceptualization, Resources |  |  |
| Scott | Ritchie | Resources, Data Curation, Writing – Review & Editing |  | S.C.R is funded by the National Institute for Health Research [Cambridge Biomedical Research Centre at the Cambridge University Hospitals NHS Foundation Trust] [*]. *The views expressed are those of the authors and not necessarily those of the NHS, the NIHR or the Department of Health and Social Care. |
| David | Roberts | Conceptualization, Resources, Supervision, Writing – Review & Editing |  | D.J.R. was supported by the NIHR Programme "Erythropoiesis in Health and Disease" (NIHR-RP-PG-0310-1004). |
| Benjamin | Rodriguez | Conceptualization, Resources |  |  |
| Jonathan | Rosen | Conceptualization, Resources |  |  |
| Jerome | Rotter | Conceptualization, Resources |  | Support for the statistical analyses in MESA was provided by R01 HL120393 and R01 HL105756. MESA and the MESA SHARe projects are conducted and supported by the National Heart, Lung, and Blood Institute (NHLBI) in collaboration with MESA investigators. Support for MESA is provided by contracts HHSN268201500003I, N01-HC-95159, N01-HC-95160, N01-HC-95161, N01-HC-95162, N01-HC-95163, N01-HC-95164, N01-HC-95165, N01-HC-95166, N01-HC-95167, N01-HC-95168, N01-HC-95169, UL1-TR-000040, UL1-TR-001079, UL1-TR-001420. Also supported in part by the National Center for Advancing Translational Sciences, CTSI grant UL1TR001881, and the National Institute of Diabetes and Digestive and Kidney Disease Diabetes Research Center (DRC) grant DK063491. Funding for SHARe genotyping was provided by NHLBI Contract N02-HL-64278. Genotyping was performed at Affymetrix (Santa Clara, California, USA) and the Broad Institute of Harvard and MIT (Boston, Massachusetts, USA) using the Affymetrix Genome-Wide Human SNP Array 6.0. |
| Saori | Sakaue |  |  |  |
| Vijay | Sankaran | Conceptualization, Resources, Supervision, Writing – Original Draft, Funding Acquisition |  | V.G.S. is supported by NIH grants R01 DK103794, a gift from the Lodish Family to Boston Children's Hospital, and the New York Stem Cell Foundation (NYSCF). V.G.S. is a NYSCF-Robertson Investigator. |
| Petra | Schubert | Conceptualization, Resources |  |  |
| Nicole | Soranzo | Conceptualization, Resources, Supervision, Writing – Original Draft, Funding Acquisition |  | NS is supported by the Wellcome Trust [grant number 206194], by the NIHR Cambridge Biomedical Research Centre Biomedical Resources Grant, University of Cambridge, Cardiovascular Theme [RG64226], by the NIHR Blood and Transplant Research Unit [NIHR BTRU-2014-10024] and by the Cambridge BHF Centre of Research Excellence [RE/18/1/34212]. |
| Cassandra | Spracklen | Conceptualization, Resources |  | CNS was supported by American Heart Association Postdoctoral Fellowship 15POST24470131 and 17POST33650016 |
| Oliver | Stegle | Conceptualization, Resources |  |  |
| Praveen | Surendran | Conceptualization, Resources, Formal Analysis |  | PS is funded by a Rutherford Fund Fellowship from the Medical Research Council grant MR/S003746/1 |
| Hua | Tang | Conceptualization, Resources |  |  |
| Manuel | Tardaguila | Conceptualization, Methodology, Software, Formal Analysis, Writing – Review & Editing |  |  |
| Jean-Claude | Tardif | Conceptualization, Resources |  |  |
| Frank | van Rooij | Conceptualization, Resources |  | The Rotterdam Study is funded by Erasmus Medical Center and Erasmus University, Rotterdam, Netherlands Organization for the Health Research and Development (ZonMw), the Research Institute for Diseases in the Elderly (RIDE), the Ministry of Education, Culture and Science, the Ministry for Health, Welfare and Sports, the European Commission (DG XII), and the Municipality of Rotterdam. The authors are grateful to the study participants, the staff from the Rotterdam Study and the participating general practitioners and pharmacists. |
| Uwe | Völker | Conceptualization, Resources |  | SHIP is part of the Community Medicine Research net of the University of Greifswald, Germany (www.community-medicine.de), which is funded by the Federal Ministry of Education and Research (grants no. 01ZZ9603, 01ZZ0103, and 01ZZ0403), the Siemens AG, the Ministry of Cultural Affairs as well as the Social Ministry of the Federal State of Mecklenburg-West Pomerania, and the network ‘Greifswald Approach to Individualized Medicine (GANI_MED)’ funded by the Federal Ministry of Education and Research (grant 03IS2061A). ExomeChip data have been supported by the Federal Ministry of Education and Research (grant no. 03Z1CN22) and the Federal State of Mecklenburg-West Pomerania. |
| Henry | Völzke | Conceptualization, Resources |  | SHIP is part of the Community Medicine Research net of the University of Greifswald, Germany (www.community-medicine.de), which is funded by the Federal Ministry of Education and Research (grants no. 01ZZ9603, 01ZZ0103, and 01ZZ0403), the Siemens AG, the Ministry of Cultural Affairs as well as the Social Ministry of the Federal State of Mecklenburg-West Pomerania, and the network ‘Greifswald Approach to Individualized Medicine (GANI_MED)’ funded by the Federal Ministry of Education and Research (grant 03IS2061A). ExomeChip data have been supported by the Federal Ministry of Education and Research (grant no. 03Z1CN22) and the Federal State of Mecklenburg-West Pomerania. |
| Dragana | Vuckovic | Conceptualization, Methodology, Software, Formal Analysis, Writing – Original Draft |  | DV is funded by the National Institute for Health Research Blood and Transplant Research Unit in Donor Health and Genomics (NIHR BTRU-2014-10024). |
| Klaudia | Walter | Conceptualization, Resources |  |  |
| Nicholas | Watkins | Conceptualization, Resources |  |  |
| Stephen | Watt | Conceptualization, Resources |  |  |
| Stefan | Weiss | Conceptualization, Resources |  | SHIP is part of the Community Medicine Research net of the University of Greifswald, Germany (www.community-medicine.de), which is funded by the Federal Ministry of Education and Research (grants no. 01ZZ9603, 01ZZ0103, and 01ZZ0403), the Siemens AG, the Ministry of Cultural Affairs as well as the Social Ministry of the Federal State of Mecklenburg-West Pomerania, and the network ‘Greifswald Approach to Individualized Medicine (GANI_MED)’ funded by the Federal Ministry of Education and Research (grant 03IS2061A). ExomeChip data have been supported by the Federal Ministry of Education and Research (grant no. 03Z1CN22) and the Federal State of Mecklenburg-West Pomerania. |
| Emilie | Wigdor | Resources, Data Curation, Writing – Review & Editing |  |  |
| Peter | Wilson | Conceptualization, Resources |  |  |
| Alan | Zonderman | Conceptualization, Resources |  |  |
|  |  |  |  |  |

**VA Million Veteran Program membership**

MVP Executive Committee

• Co-Chair: J. Michael Gaziano, M.D., M.P.H.

• Co-Chair: Rachel Ramoni, D.M.D., Sc.D.

• Jim Breeling, M.D. (ex-officio)

• Kyong-Mi Chang, M.D.

• Grant Huang, Ph.D.

• Sumitra Muralidhar, Ph.D.

• Christopher J. O’Donnell, M.D., M.P.H.

• Philip S. Tsao, Ph.D.

MVP Program Office

• Sumitra Muralidhar, Ph.D.

• Jennifer Moser, Ph.D.

MVP Recruitment/Enrollment

• Recruitment/Enrollment Director/Deputy Director, Boston – Stacey B. Whitbourne, Ph.D.; Jessica V. Brewer, M.P.H.

• MVP Coordinating Centers

- Clinical Epidemiology Research Center (CERC), West Haven – John Concato, M.D., M.P.H.

- Cooperative Studies Program Clinical Research Pharmacy Coordinating Center, Albuquerque - Stuart Warren, J.D., Pharm D.; Dean P. Argyres, M.S.

- Genomics Coordinating Center, Palo Alto – Philip S. Tsao, Ph.D.

- Massachusetts Veterans Epidemiology Research Information Center (MAVERIC), Boston - J. Michael Gaziano, M.D., M.P.H.

- MVP Information Center, Canandaigua – Brady Stephens, M.S.

• Core Biorepository, Boston – Mary T. Brophy M.D., M.P.H.; Donald E. Humphries, Ph.D.

• MVP Informatics, Boston – Nhan Do, M.D.; Shahpoor Shayan

• Data Operations/Analytics, Boston – Xuan-Mai T. Nguyen, Ph.D.

MVP Science

• Genomics - Christopher J. O’Donnell, M.D., M.P.H.; Saiju Pyarajan Ph.D.; Philip S. Tsao, Ph.D.

• Phenomics - Kelly Cho, M.P.H, Ph.D.

• Data and Computational Sciences – Saiju Pyarajan, Ph.D.

• Statistical Genetics – Elizabeth Hauser, Ph.D.; Yan Sun, Ph.D.; Hongyu Zhao, Ph.D.

MVP Local Site Investigators

• Atlanta VA Medical Center (Peter Wilson)

• Bay Pines VA Healthcare System (Rachel McArdle)

• Birmingham VA Medical Center (Louis Dellitalia)

• Cincinnati VA Medical Center (John Harley)

• Clement J. Zablocki VA Medical Center (Jeffrey Whittle)

• Durham VA Medical Center (Jean Beckham)

• Edith Nourse Rogers Memorial Veterans Hospital (John Wells)

• Edward Hines, Jr. VA Medical Center (Salvador Gutierrez)

• Fayetteville VA Medical Center (Gretchen Gibson)

• VA Health Care Upstate New York (Laurence Kaminsky)

• New Mexico VA Health Care System (Gerardo Villareal)

• VA Boston Healthcare System (Scott Kinlay)

• VA Western New York Healthcare System (Junzhe Xu)

• Ralph H. Johnson VA Medical Center (Mark Hamner)

• Wm. Jennings Bryan Dorn VA Medical Center (Kathlyn Sue Haddock)

• VA North Texas Health Care System (Sujata Bhushan)

• Hampton VA Medical Center (Pran Iruvanti)

• Hunter Holmes McGuire VA Medical Center (Michael Godschalk)

• Iowa City VA Health Care System (Zuhair Ballas)

• Jack C. Montgomery VA Medical Center (Malcolm Buford)

• James A. Haley Veterans’ Hospital (Stephen Mastorides)

• Louisville VA Medical Center (Jon Klein)

• Manchester VA Medical Center (Nora Ratcliffe)

• Miami VA Health Care System (Hermes Florez)

• Michael E. DeBakey VA Medical Center (Alan Swann)

• Minneapolis VA Health Care System (Maureen Murdoch)

• N. FL/S. GA Veterans Health System (Peruvemba Sriram)

• Northport VA Medical Center (Shing Shing Yeh)

• Overton Brooks VA Medical Center (Ronald Washburn)

• Philadelphia VA Medical Center (Darshana Jhala)

• Phoenix VA Health Care System (Samuel Aguayo)

• Portland VA Medical Center (David Cohen)

• Providence VA Medical Center (Satish Sharma)

• Richard Roudebush VA Medical Center (John Callaghan)

• Salem VA Medical Center (Kris Ann Oursler)

• San Francisco VA Health Care System (Mary Whooley)

• South Texas Veterans Health Care System (Sunil Ahuja)

• Southeast Louisiana Veterans Health Care System (Amparo Gutierrez)

• Southern Arizona VA Health Care System (Ronald Schifman)

• Sioux Falls VA Health Care System (Jennifer Greco)

• St. Louis VA Health Care System (Michael Rauchman)

• Syracuse VA Medical Center (Richard Servatius)

• VA Eastern Kansas Health Care System (Mary Oehlert)

• VA Greater Los Angeles Health Care System (Agnes Wallbom)

• VA Loma Linda Healthcare System (Ronald Fernando)

• VA Long Beach Healthcare System (Timothy Morgan)

• VA Maine Healthcare System (Todd Stapley)

• VA New York Harbor Healthcare System (Scott Sherman)

• VA Pacific Islands Health Care System (Gwenevere Anderson)

• VA Palo Alto Health Care System (Philip Tsao)

• VA Pittsburgh Health Care System (Elif Sonel)

• VA Puget Sound Health Care System (Edward Boyko)

• VA Salt Lake City Health Care System (Laurence Meyer)

• VA San Diego Healthcare System (Samir Gupta)

• VA Southern Nevada Healthcare System (Joseph Fayad)

• VA Tennessee Valley Healthcare System (Adriana Hung)

• Washington DC VA Medical Center (Jack Lichy)

• W.G. (Bill) Hefner VA Medical Center (Robin Hurley)

• White River Junction VA Medical Center (Brooks Robey)

• William S. Middleton Memorial Veterans Hospital (Robert Striker)
